# Identifying the Cognitive Processes Underpinning Hippocampal-Dependent Tasks

**DOI:** 10.1037/xge0000582

**Published:** 2019-03-04

**Authors:** Ian A. Clark, Victoria Hotchin, Anna Monk, Gloria Pizzamiglio, Alice Liefgreen, Eleanor A. Maguire

**Affiliations:** 1 Wellcome Centre for Human Neuroimaging, Queen Square Institute of Neurology, University College London

**Keywords:** autobiographical memory, future thinking, navigation, scene construction, individual differences

## Abstract

Autobiographical memory, future thinking, and spatial navigation are critical cognitive functions that are thought to be related and are known to depend upon a brain structure called the hippocampus. Surprisingly, direct evidence for their interrelatedness is lacking, as is an understanding of why they might be related. There is debate about whether they are linked by an underlying memory-related process or, as has more recently been suggested, because they each require the endogenous construction of scene imagery. Here, using a large sample of participants and multiple cognitive tests with a wide spread of individual differences in performance, we found that these functions are indeed related. Mediation analyses further showed that scene construction, and not memory, mediated (explained) the relationships between the functions. These findings offer a fresh perspective on autobiographical memory, future thinking, navigation, and also on the hippocampus, where scene imagery appears to play an influential role.

Our past experiences are captured in autobiographical memories that serve to sustain our sense of self, enable independent living, and prolong survival (Tulving, 2002). Consequently, a key aim of cognitive psychology and neuropsychology has been to understand how such memories are formed and recollected. There is wide agreement that a brain structure called the *hippocampus* plays a key role in supporting autobiographical memories. Patients with hippocampal damage are impaired at recalling past experiences (Scoville & Milner, 1957; see also Clark & Maguire, 2016; Verfaellie & Keane, 2017; Winocur & Moscovitch, 2011), and the hippocampus is consistently engaged during functional MRI studies of autobiographical memory retrieval (Cabeza & St. Jacques, 2007; Svoboda, McKinnon, & Levine, 2006). Consequently, the hippocampus and autobiographical memory have become synonymous.

However, the hippocampus has been associated with functions beyond autobiographical memory. The animal literature has, for many years, placed spatial navigation at the heart of hippocampal processing (Moser, Kropff, & Moser, 2008; O’Keefe & Dostrovsky, 1971; O’Keefe & Nadel, 1978), with concordant findings in humans (Ekstrom et al., 2003; Epstein, Patai, Julian, & Spiers, 2017; Maguire et al., 2000). Work over the past decade has also linked the hippocampus with thinking about the future (Addis, Wong, & Schacter, 2007; Hassabis, Kumaran, Vann, & Maguire, 2007), the imagination of scenes and events (Hassabis, Kumaran, Vann, et al., 2007; Schacter et al., 2012), the perception of scenes (Graham, Barense, & Lee, 2010; McCormick, Rosenthal, Miller, & Maguire, 2017), and specific aspects of visuospatial processing, including perceptual richness, a sense of reliving and imagery content (Andrews-Hanna, Reidler, Sepulcre, Poulin, & Buckner, 2010; St-Laurent, Moscovitch, & McAndrews, 2016; St. Jacques, Conway, Lowder, & Cabeza, 2010).

The link between autobiographical memory, the construction of scene imagery in the imagination (scene construction), and thinking about the future has come under increasing scrutiny. Studies of amnesic patients have reported deficits in tasks assessing each of these functions (Hassabis, Kumaran, Vann, et al., 2007; Klein, Loftus, & Kihlstrom, 2002; Rosenbaum et al., 2005; Tulving, 1985). In neuroimaging studies, the recruitment of the same neural network, including the hippocampus, has been observed when thinking about the past, the future, or atemporal events and scenes with no obvious focus in time (Buckner & Carroll, 2007; Hassabis & Maguire, 2007; Schacter, Addis, & Buckner, 2007). In addition, comparisons of behavioral measures have highlighted similarities in terms of ratings of vividness and the amount and type of details for past, future, and atemporal events (D’Argembeau & Van der Linden, 2006; de Vito, Gamboz, & Brandimonte, 2012). Overall, therefore, autobiographical memory, scene construction, and thinking about the future seem to involve the hippocampus, with parallels also in the pattern of behavioral outcomes. Yet, conceptually, they are different processes not least in terms of the temporal context within which the scene or event is imagined. The question, therefore, arises as to what does the hippocampus do in the service of each of these functions?

One suggestion is that autobiographical memory provides the building blocks for thinking about the future and imagining atemporal scenes and events and, as such, their dependence on the hippocampus is fundamentally mnemonic (Moscovitch, Cabeza, Winocur, & Nadel, 2016; Schacter et al., 2012; Sheldon & Levine, 2016). This is based upon the suggestion that autobiographical memory recall is a constructive process that recombines different elements to recreate memories (e.g., Schacter et al., 2012). This information is also available for the construction of nonautobiographical memory events. In this regard, the autobiographical memory system is equally well equipped to imagine future or atemporal events as well as recalling the past (see also St. Jacques, Carpenter, Szpunar, & Schacter, 2018; Thakral, Benoit, & Schacter, 2017).

An alternative view is that the mental construction of scene imagery is a key process that autobiographical memory, future thinking, and spatial navigation have in common (Maguire & Mullally, 2013; see also Robin, 2018, and Rubin & Umanath, 2015, for related theoretical viewpoints). A scene is a naturalistic three-dimensional spatially coherent representation of the world typically populated by objects and viewed from an egocentric perspective. When most people recall the past, imagine the future or plan a route during navigation, scenes feature prominently. An individual’s ability to use scene imagery, or spatial context, to imagine or recall an event, has been shown to predict the vividness and detail of the imagined scenario (Arnold, McDermott, & Szpunar, 2011; D’Argembeau & Van der Linden, 2004; Hebscher, Levine, & Gilboa, 2017; Robin & Moscovitch, 2014; Robin, Wynn, & Moscovitch, 2016; Sheldon & Chu, 2017; Szpunar & McDermott, 2008). Furthermore, damage limited to the hippocampus is known to impede the ability to construct endogenous scene imagery (Andelman, Hoofien, Goldberg, Aizenstein, & Neufeld, 2010; Hassabis, Kumaran, Vann, et al., 2007; Maguire & Mullally, 2013; Race, Keane, & Verfaellie, 2011; Rosenbaum, Gilboa, Levine, Winocur, & Moscovitch, 2009). The mental construction of scenes is, therefore, both reliant upon hippocampal functionality and related to autobiographical memory, future thinking, and spatial navigation.

There is, however, a dearth of evidence available that permits adjudication between a mnemonic or scene construction account of hippocampal function. Arguably, extant evidence highlights the importance of scene construction over autobiographical memory (de Vito et al., 2012; Palombo, Hayes, Peterson, Keane, & Verfaellie, 2018; Robin & Moscovitch, 2014; but see also, Addis, Cheng, Roberts, & Schacter, 2011; Roberts, Schacter, & Addis, 2017). To the best of our knowledge, there are no large-scale individual differences studies systematically examining the direct relationships between scene construction, autobiographical memory, thinking about the future and spatial navigation.

We, therefore, had two overarching goals in the current study. First, we sought to investigate whether scores on tasks assessing scene construction, autobiographical memory, future thinking, and spatial navigation were related. Second, if they were related, we wished to unpack these relationships further by attempting to pinpoint whether the link between them could be best explained by either a scene construction or an autobiographical memory process. Given that the scene construction deficit of hippocampal-damaged patients is evident even on nonmnemonic tasks, for example, the visual perception of scenes (Lee et al., 2005; McCormick et al., 2017), we hypothesized that the relationship, if any, between the tasks would be best explained by scene construction rather than by autobiographical memory.

To address our first goal, we conducted a principal component analysis (PCA) involving a large range of cognitive tests. This allowed us to assess whether or not performance on tasks examining scene construction, autobiographical memory, future thinking, and navigation was related in the presence of other cognitive tasks.

To pursue our second goal, we performed a series of mediation analyses. This approach allowed us to investigate how scores on the tasks of interest were related. Mediation analyses focus on whether one variable can explain (mediate) the relationship between two different variables. In short, we expected that if the cognitive process linking the different tasks together was related to scenes, then scene construction performance would mediate the task relationships. By contrast, if the underlying process was related to autobiographical memory, then autobiographical memory performance would be found to mediate.

We recruited a large group of participants and assessed their performance on a comprehensive battery of cognitive tasks, including measures of scene construction, autobiographical memory, future thinking, and navigation. Tasks were chosen from the published literature because of their confirmed reliance (or nonreliance) upon the hippocampus.

## Method

### Participants

Two hundred and seventeen individuals were recruited. They were aged between 20 and 41 years old, had English as their first language and reported no psychological, psychiatric, neurological, or behavioral health conditions. The age range was restricted to 20–41 to limit the possible effects of ageing. Participants reporting hobbies or vocations known to be associated with the hippocampus (e.g., licensed London taxi drivers) were excluded. The mean age of the sample was 29.0 years (95% confidence interval [CI]: 20, 38) and included 109 women and 108 men. Participants were reimbursed £10 per hour for taking part which was paid at study completion. All participants gave written informed consent and the study was approved by the University College London Research Ethics Committee. American Psychological Association ethical standards were complied with in regards to the treatment of the participants.

The sample size was determined at 216 during study design to be robust to employing different statistical approaches when answering multiple questions of interest. Specifically, the sample allows for sufficient power to identify medium effect sizes when conducting regression analyses, which form the basis of mediation analyses, at alpha levels of .01 (Cohen, 1992). Importantly, the sample size is also large enough to conduct mediation analyses and structural equation modeling (Anderson & Gerbing, 1988). A final sample of 217 was obtained due to over recruitment.

### Procedure

Participants completed the study over three separate visits. The order of tests within each visit was the same for all participants (see the Task Order section). Task order was arranged so as to avoid task interference, for example, not having a verbal test followed by another verbal test, and to provide sessions of approximately equal length (∼3–3.5 hr, including breaks). All participants completed all parts of the study.

### Cognitive Tests

#### Measures of primary interest

Our main interest was in scene construction, autobiographical memory, future thinking, and navigation; tasks that are known to recruit or require the hippocampus to be successfully completed. All tasks are published and were performed and scored as per their published use. Given the extensive task battery that was used, only the main outcome measure was used for each task to reduce potential issues surrounding multiple comparisons and false positives. Here, for the reader’s convenience, we describe each task briefly.

##### Scene construction test

The scene construction test (Hassabis, Kumaran, Vann, et al., 2007) measures a participant’s ability to mentally construct a visual scene. Participants construct different scenes of commonplace settings. For each scene, a short cue is provided (e.g., imagine lying on a beach in a beautiful tropical bay) and the participant is asked to imagine the scene that is evoked and then describe it out loud in as much detail as possible. Recordings are transcribed for later scoring. Participants are explicitly told not to describe a memory, but to create a new scene that they have never experienced before.

The overall outcome measure is an “experiential index” that is calculated for each scene and then averaged. In brief, it is composed of four elements: the content, participant ratings of their sense of presence (how much they felt like they were really there) and perceived vividness, participant ratings of the spatial coherence of the scene, and an experimenter rating of the overall quality of the scene.

Double scoring was performed on 20% of the data. We took the most stringent approach to identifying across-experimenter agreement. Interclass correlation coefficients, with a two-way random effects model looking for absolute agreement indicated excellent agreement among the experimenter ratings (minimum score of .9; see Supplemental Table S1 in the online supplementary materials). For reference, a score of .8 or above is considered excellent agreement beyond chance.

##### Autobiographical interview

In the autobiographical interview (AI; Levine, Svoboda, Hay, Winocur, & Moscovitch, 2002) participants are asked to provide autobiographical memories from a specific time and place over four time periods—early childhood (up to age 11), teenage years (aged from 11–17), adulthood (from age 18 years to 12 months prior to the interview; two memories are requested), and the last year (a memory from the last 12 months). Recordings are transcribed for later scoring.

In contrast to the other tasks, the AI has two main outcome measures, both of which are consistently reported in the literature. Memories are scored to collect “internal” and “external” details of the event. Importantly, these two scores represent different aspects of autobiographical memory recall. Internal details are those describing the event in question (i.e., episodic details). External details describe semantic information concerning the event, or nonevent information. Internal events are therefore thought to be hippocampal-dependent, while external events are not. As such, in line with the published literature, we report both outcome measures. The two AI scores are obtained by separately averaging performance for the internal and external details across five autobiographical memories. Our double scoring produced excellent agreement across the experimenters (minimum score of .81; see Supplemental Table S2 in the online supplementary materials).

##### Future thinking test (Hassabis, Kumaran, Vann, et al., 2007)

This test follows the same procedure as the scene construction test but requires participants to imagine three plausible future scenes involving themselves (an event at the weekend; next Christmas; the next time they will meet a friend). Participants are explicitly told not to describe a memory, but to create a new future scene. Recordings are transcribed for later scoring. The scoring procedures are the same as for scene construction. Double scoring identified excellent agreement across the experimenters (minimum score of .88; see Supplemental Table S3 in the online supplementary materials).

##### Navigation tests (Woollett & Maguire, 2010)

Navigation ability is assessed using movies of navigation through an unfamiliar town. Movie clips of two overlapping routes through this real town (Blackrock, in Dublin, Ireland) are shown to participants four times.

Five tasks are used to assess navigational ability. First, following each viewing of the route movies, participants are shown four short clips—two from the actual routes, and two distractors. Participants indicate whether they have seen each clip or not. Second, after all four route viewings are completed, recognition memory for scenes from the routes is tested. A third test involves assessing knowledge of the spatial relationships between landmarks from the routes. Fourth, route knowledge is examined by having participants place photographs from the routes in the correct order as if traveling through the town. Finally, participants draw a sketch map of the two routes including as many landmarks as they can remember. Sketch maps are scored in terms of the number of road segments, road junctions, correct landmarks, landmark positions, the orientation of the routes and an overall map quality score from the experimenters. Double scoring was performed on 20% of the sketch maps finding excellent agreement (minimum of .89; see Supplemental Table S4 in the online supplementary materials). An overall navigation score is calculated by combining scores from all of the above tasks.

#### Additional measures

We administered a range of other tasks to participants which enabled us to further profile their cognition. In brief, estimates of IQ were obtained using the Test of Premorbid Functioning (TOPF; Wechsler, 2011). The number of correct responses was converted to an estimate of Full Scale IQ (FSIQ) as per the TOPF scoring procedure. General intellect and executive functioning were measured using the Matrix Reasoning subtest of the Wechsler Adult Intelligence Scale IV (Wechsler, 2008), the Brixton Spatial Anticipation Test (Burgess & Shallice, 1997) and the F-A-S verbal fluency task (F-A-S; Strauss, Sherman, & Spreen, 2006). Working memory/attention was assessed using the Digit Span subtest of the Wechsler Adult Intelligence Scale IV and the Symbol Span subtest of the Wechsler Memory Scale IV (Wechsler, 2009).

Visuospatial recall was examined using the Rey–Osterrieth Complex Figure (ROCF; Rey, 1941). In addition, we also used an object-place association test which required participants to learn the locations of 16 objects presented simultaneously on a white computer screen (adapted from Woollett & Maguire, 2009). The outcome measure was the number of trials (maximum of 6) taken to correctly learn the location of all the objects, with a score of seven if the array was never learnt (this was reverse scored for ease of interpretation with the other tasks).

Verbal recall was assessed using the Rey Auditory Verbal Learning Test (RAVLT; see Strauss et al., 2006), and the Logical Memory and Verbal Paired Associates subtests of the Wechsler Memory Scale IV (Wechsler, 2009). Two additional verbal recall tasks were also included (Clark, Kim, & Maguire, 2018). A limitation of the Wechsler Memory Scale Verbal Paired Associates task is its reliance on concrete, imageable words (Clark & Maguire, 2016; Maguire & Mullally, 2013). We therefore included two additional versions of this task. In one case, only concrete, imageable words are used, whereas the other comprises only abstract, nonimageable words. The two tests are precisely matched apart from the imageability of the words. For all of these recall tasks, the delayed recall scores were used as our primary data as they are most sensitive to hippocampal damage (Squire, 1992).

Recognition memory was assessed using the Warrington Recognition Memory Tests for words, faces, and scenes (Cipolotti & Maguire, 2003; Warrington, 1984). Semantic memory was assessed using the “Dead or Alive” task which probes general knowledge about whether famous individuals have died or are still alive (Kapur, Young, Bateman, & Kennedy, 1989).

General visuospatial processing was assessed using the Paper Folding Test (Ekstrom, French, Harman, & Dermen, 1976), which measures a participant’s ability to transform images of spatial patterns into different arrangements. Perceptual processing was assessed using scene description and boundary extension tasks (Mullally, Intraub, & Maguire, 2012). The scene description task requires participants to describe a picture of a scene. The content of participants’ descriptions is scored across a number of categories and summed to provide a total content score. Double scoring was performed on 20% of the descriptions finding excellent agreement (minimum of .85; see Supplemental Table S5 in the online supplementary materials). Boundary extension occurs when individuals who are viewing scenes automatically imagine what might be beyond the view, and consequently later misremember having seen a greater expanse of the scene (Intraub & Richardson, 1989). To test this, participants are briefly presented on each trial with two pictures in rapid succession and are asked to rate whether the second picture is of a closer perspective (when boundary extension is induced), exactly the same (the correct answer), or further away. Unbeknownst to participants, the majority of images are exactly the same. The outcome measure was the proportion of same trials classed as closer-up.

#### Task order

The tests were conducted in the following order: In Session 1, Concrete Verbal Paired Associates (learning), Warrington Recognition Memory Test for scenes, Dead or Alive Task, Symbol Span Test, Scene Description Task, Concrete Verbal Paired Associates (delayed recall), Logical Memory test (learning), ROCF (copy), TOPF, Warrington Recognition Memory Test for faces, Brixton Spatial Anticipation Test, Logical Memory test (delayed recall), ROCF (delayed recall), and Warrington Recognition Memory Test for words. In Session 2, navigation tests, followed by Abstract Verbal Paired Associates (learning, with delayed recall 30 min later). In Session 3, Scene Construction Test, Future Thinking Test, RAVLT (learning), Paper Folding Test, Digit Span Test, Matrix Reasoning, RAVLT (delayed recall), Autobiographical Interview, Wechsler Memory Scale Verbal Paired Associates (learning), object-place association test, boundary extension task, Wechsler Memory Scale Verbal Paired Associates (delayed recall), and F-A-S task.

### Statistical Analyses

Data are summarized using means and 95% CIs, calculated in SPSS v22. PCA was performed using SPSS v22, with varimax rotation and a cut-off at an eigenvalue of 1. Regression analyses with standardized beta values and confidence intervals were performed in R v3.4. Mediation and sensitivity analyses were performed using the R Causal Mediation Analysis package v4.4.6 (Imai, Keele, & Tingley, 2010). Structural equation modeling (SEM) was performed using the R Lavaan package v0.6–1.1178 (Rosseel, 2012) and assessed for model fit as per the criteria of Hu and Bentler (1999). Effect sizes are reported as *R*^2^ values for regressions, including those regressions used in the mediation and SEM analyses (adjusted *R*^2^ when multiple variables were included) and as sensitivity analyses for the mediation analyses. There were no missing data, and no data needed to be removed from any analysis.

## Results

A summary of the outcome measures for the cognitive tasks is presented in Table 1. A wide range of scores was obtained for all variables.[Table-anchor tbl1]

### How Are the Tasks Interrelated?

We first asked whether performance across the tasks of primary interest (scene construction, autobiographical memory, future thinking and navigation), was related. If, in line with our prediction, these tasks share an underlying cognitive process, then performance on one task should be related to performance on the others. More generally, we also sought to investigate this within the wider context of the other cognitive tasks.

We performed a PCA using all of the tasks in order to avoid selection bias—we had no reason to exclude any task from the PCA. Varimax rotation was applied (to allow for cross-over between the derived components) and the minimum eigenvalue was set to 1. The PCA identified seven components that explained 59.24% of the variance. Examination of the Scree plot supported the seven component solution (see the online supplementary materials and Supplemental Figure S1).

Naming of the components was determined by the tasks that most strongly loaded on to each (see Table 2 for the proportion of variance explained by each component, Table 3 for the tasks in each component and their weightings, and Supplemental Table S6 in the online supplementary materials for all weightings). Component 1 comprised tasks with a particularly strong spatial component (e.g., navigation, object-place association, paper folding). Notably, this was regardless of whether or not memory was required; for example, the Paper Folding Test and the Brixton Spatial Anticipation Test had minimal memory requirements. Component 2 contained all of the verbal memory tasks. Component 3 comprised those tasks typically thought to assess general IQ or executive function. Matrix Reasoning and the estimate of FSIQ from the TOPF are designed to be measures of general IQ (Wechsler, 2008, 2011), the Symbol Span and Digit Span Tests measure executive function, working memory, and attention (Wechsler, 2008, 2009), and the F-A-S is reported as an executive function task (Strauss et al., 2006). Finally, although the Abstract Verbal Paired Associates test is a verbal memory task (and aligns also with Component 2), it is a more challenging task than the other verbal memory tasks—as shown by the performance scores in Table 1—and has also been found to require the recruitment of frontal “executive” brain regions (Clark et al., 2018), suggesting that processing of abstract verbal paired associates may reflect general IQ as well as verbal memory. Component 4 involved three of our tasks of primary interest: scene construction, autobiographical memory (internal details) and future thinking, and the inclusion of the simple scene description task (which also loaded onto the perceptual component). For convenience, we refer to this component as the *scene component*, as per our hypothesis that these tasks have scenes in common but acknowledge that this remains to be tested in our following analyses. Component 5 contained the three recognition memory tests and Component 6 the two semantic tasks. Finally, Component 7, while also using scene-based stimuli, contained the two tasks that primarily assessed visual perception.[Table-anchor tbl2][Table-anchor tbl3]

There are, however, two potential limitations to the PCA that should be noted. First, it could be suggested that inclusion of FSIQ in the PCA reduces the pool of domain-specific variance that can be partitioned because FSIQ could be regarded as a more nonspecific cognitive task. To ensure FSIQ scores were not biasing the results, we performed the PCA again without the FSIQ scores, finding a near identical pattern of results (see Supplemental Table S7 in the online supplementary materials). The only notable effect of removing FSIQ was that Components 3 and 4 were switched, with the scene component now explaining a greater proportion of the variance than the IQ/executive function component.

Second, the selection of the number of components from a PCA is, at least in part, a subjective decision. From the scree plot, it could be argued that our PCA should only result in four factors, rather than seven. However, as has been widely discussed in the literature, determining the number of components to include requires the balancing of parsimony and plausibility (see, e.g., Fabrigar, Wegener, MacCallum, & Strahan, 1999), with the selection of too few components being typically regarded as a much more severe error than too many (Cattell, 1978; Fava & Velicer, 1992; Rummel, 1970; Wood, Tataryn, & Gorsuch, 1996). As detailed in the >online supplementary materials, restricting the model to four components meant that two tasks (Dead or Alive, Boundary Extension) loaded onto none of the components. Furthermore, limiting the solution to four components led to the loss of the recognition memory, semantic memory, and perception components, resulting, for example, in the Warrington Recognition Memory Test for faces loading onto the verbal memory component. As such, specifying only four components obscured theoretically plausible and relevant components as well as leading to difficulties in interpretation. On the other hand, the seven-component solution was statistically valid, created a clear factor structure with all the tests included, and all components had both statistical and theoretical value.

We also note that our main focus here was on scene construction, autobiographical memory, future thinking and navigation, and investigating how performance on these tasks is related in the presence of other cognitive tasks. Regardless of the above issues—the inclusion/exclusion of FSIQ and the selection of either four or seven components—our main tasks of interest followed the same loadings and the scene and spatial components remained within the top four explanatory components.

In summary, performance on the scene construction, autobiographical memory (internal details), and future thinking tasks all aligned onto the same component. This demonstrates that these tasks are strongly related in cognitive terms. However, surprisingly, the navigation task did not load onto this component, but instead loaded onto the spatial component—a point we will return to later.

Although the PCA can tell us about the main relationships between tasks, it cannot inform about the nature of the underlying processes. We therefore proceeded to perform additional analyses to examine this.

### What Cognitive Process(es) Underpin the Scene Component?

The PCA analysis identified that, with the exception of navigation, our tasks of primary interest—scene construction, autobiographical memory internal details (henceforth referred to as autobiographical memory), and future thinking—all loaded onto one component—scenes. As the scene description task also loaded onto the perception component as well as the scene component, it was not included in the following analyses to allow for the assessment of just the pure elements of the scene component.

We used mediation analyses to investigate possible processes underpinning the scene component. This method aims to explain the mechanisms and/or processes underlying the relationship between two variables via the inclusion of a third variable. If the third variable fully mediates the original relationship, this provides evidence that the link between the original variables can be explained solely due to the mediating variable. This is known as an indirect effect. On the other hand, if no indirect effect is identified, leaving only the direct relationship between the original variables, it can be concluded that the mediating variable is not involved in the original relationship. For a mediation analysis to be possible, there are two main requirements, as described by Baron and Kenny (1986). First, the independent variable must be a predictor of the dependent variable. Second, the independent variable must predict the mediator variable. The first requirement has, however, been further scrutinised, with some suggesting that an initial direct relationship between the independent variable and the dependent variable is not required when there is a strong a priori belief that the effect size is small or suppression is a possibility (e.g., Shrout & Bolger, 2002). However, given our substantial sample size and ability to detect small effect sizes, we followed the more stringent requirements set out by Baron and Kenny (1986) to reduce the possibility of false positives. A mediation analysis then looks at the difference between predicting the dependent variable from just the independent variable, in comparison to predicting the dependent variable from the independent variable and the mediator variable. If the relationship between the independent and dependent variable is reduced, or lost, with the inclusion of the mediator, an indirect effect has occurred.

Mediation can, therefore, be applied to our question in the following manner—if the process linking the scene component tasks is, as we hypothesize, related to scenes, the scene construction task should mediate the relationship between autobiographical memory and future thinking. Alternatively, if, as hypothesized by others, the underlying process is associated with autobiographical memory, then autobiographical memory will mediate the relationship between scene construction and future thinking. This was, therefore, our first analysis.

Before reporting the results, it is worth explaining the presentation format. A mediation analysis has two main steps. First, the initial regressions are performed to ensure mediation is possible. For ease of reading, the full details of each individual regression are reported in the >online supplementary materials and just the unstandardized coefficients are reported in the main body of the text. Second, the mediation analysis itself is performed. The mediation analysis provides two outcome measures: (a) the mediation, or indirect, effect; if this is significant there is an effect of mediation, and (b) the direct effect; if this is significant then a relationship remains between the original variables even with the inclusion of the mediator. If just the indirect effect is significant, then a full mediation has occurred. This means that all of the relationship between the independent and dependent variable can be explained by the mediator. If both the indirect and the direct effect are significant then a partial mediation has occurred. This means that some of the relationship between the independent and dependent variables can be explained by the mediator, but the independent variable still contributes to the relationship. If only the direct effect is significant, then mediation has not occurred.

In a similar manner to other statistical analyses, it is important to look not just at whether a result is significant, but how robust this effect is. For mediation, this is done via sensitivity analyses. Sensitivity analyses test how well the (indirect or direct) effect holds if additional variance is introduced into the necessary assumptions made to perform the analysis (see Imai et al., 2010). Sensitivity analyses are different from effect sizes in that there are no specific cut offs. Instead, they are used comparatively. As such, sensitivity is reported here in two forms, first, as a single value (between −1 and 1) that represents the amount of additional variance needed to reduce the effect seen to 0. A higher absolute value represents a more robust effect. Second, we also display sensitivity as a plot showing the effect of varying the additional variance on the indirect or direct effect. This allows for a visual interpretation of the robustness of the effect.

Returning to the analyses, our overarching question was whether a scene construction or an autobiographical memory process best explained the relationships identified between scene construction, autobiographical memory, and future thinking. We, therefore, systematically examined the different combinations of the relationships between these three variables. First, we investigated whether scene construction mediated the relationship between autobiographical memory and future thinking, or whether autobiographical memory mediated between scene construction and future thinking. Second, we also examined these relationships when future thinking was included as the independent instead of the dependent variable. Finally, for completeness, we investigated whether future thinking mediated the relationship between scene construction and autobiographical memory. Testing each of these in turn resulted in a complete examination of possible mediations between the three tasks.

**Figure 1 fig1:**
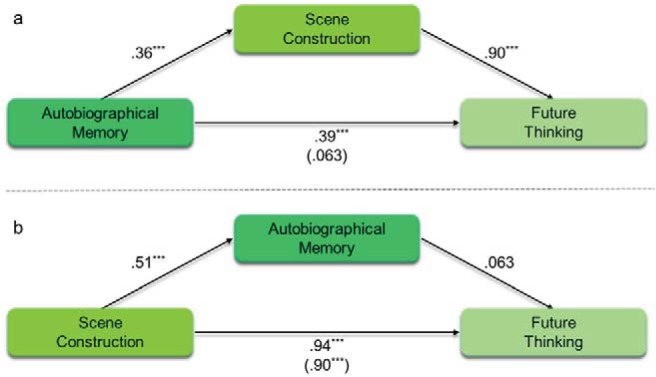
Mediation analyses of the scene component variables when future thinking is the dependent variable. (a) Autobiographical memory to future thinking, mediated by scene construction. (b) Scene construction to future thinking, mediated by autobiographical memory. The numbers in brackets show the effect of the independent variable on the dependent when the mediation variable was also taken into consideration. *** *p* < .001.

First, therefore, we sought to examine whether scene construction mediated the relationship between autobiographical memory and future thinking, and whether autobiographical memory mediated the relationship between scene construction and future thinking. The results of the mediation analyses are shown in Table 4 and Figure 1 (see Supplemental Table S8 in the online supplementary materials for the full break down of each individual regression). Figure 1a shows the relationship between autobiographical memory and future thinking, mediated by scene construction. As expected, autobiographical memory alone was associated with both future thinking (β = .39, *p* < .001) and scene construction (β = .36, *p* < .001). This shows that mediation by scene construction was possible. Indeed, with the inclusion of scene construction as a mediator, autobiographical memory was no longer related to future thinking (β = .063, *p* = .18), whereas scene construction was (β = .90, *p* < .001). Mediation analysis revealed a significant indirect effect of scene construction, with no direct effect of autobiographical memory (Table 4a). This, therefore, suggests that scene construction fully mediated (explained) the relationship between autobiographical memory and future thinking.[Table-anchor tbl4][Fig-anchor fig1]

Table 4b and Figure 1b show the equivalent analysis where autobiographical memory was placed as the mediator between scene construction and future thinking. As would be expected, the result matches the previous analysis, but with the indirect and direct effects switched. As with autobiographical memory, scene construction alone was associated with both future thinking (β = .94, *p* < .001) and autobiographical memory (β = .51, *p* < .001). This means that mediation by autobiographical memory was possible. However, including autobiographical memory as the mediator failed to show a relationship between autobiographical memory and future thinking (β = .063, *p* = .18), whereas the relationship between scene construction and future thinking remained significant (β = .90, *p* < .001). This was confirmed by the mediation analysis finding no indirect effect of autobiographical memory in comparison to the significant direct effect of scene construction. In other words, autobiographical memory could not explain the relationship between scene construction and future thinking. This contrasts with the previous analysis showing that scene construction could explain the relationship between autobiographical memory and future thinking.

**Figure 2 fig2:**
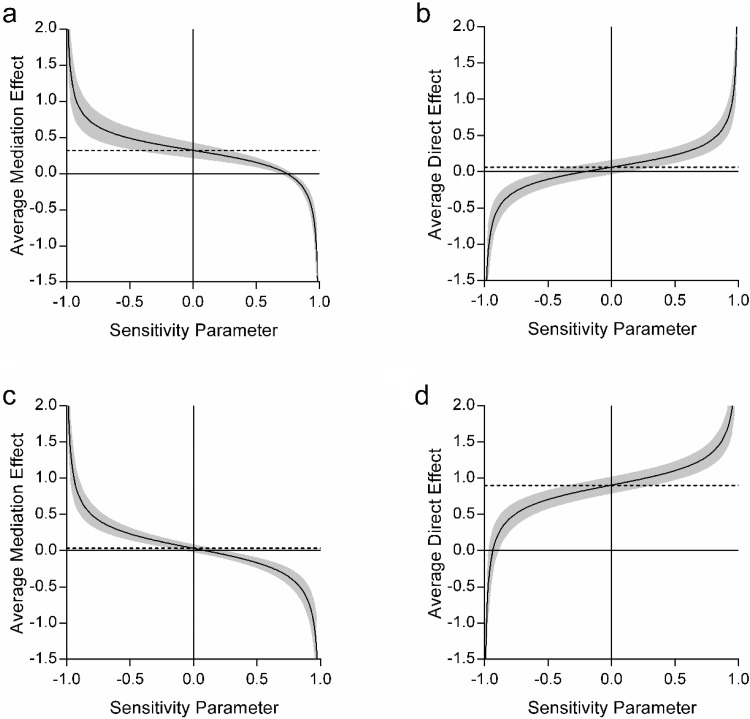
Sensitivity analyses for the indirect and direct effects of the mediation analyses of the scene component variables, when future thinking was the dependent variable. (a) Sensitivity of the indirect effect of scene construction on the relationship between autobiographical memory and future thinking. (b) Sensitivity of the direct effect between autobiographical memory and future thinking, when scene construction was taken into consideration. (c) Sensitivity of the indirect effect of autobiographical memory on the relationship between scene construction and future thinking. (d) Sensitivity of the direct effect between scene construction and future thinking, when autobiographical memory was taken into consideration. The dashed line shows the average effect when additional variance is assumed to be 0. The plotted line shows the variation in the effect when the additional variance was varied between −1 and 1 (with 95% confidence intervals). The more robust the effect, the greater the variance that was required to reduce the effect to 0 (i.e., to cross the *x*-axis).

We next performed sensitivity analyses for each of the effects (Table 4 and Figure 2). We first focused on when scene construction was the mediator between autobiographical memory and future thinking (Table 4a and Figures 2a and 2b). As can be seen from the sensitivity values, the indirect effect of scene construction (ρ = .75) was substantially more robust than the direct relationship between autobiographical memory future and thinking (ρ = −.2). On Figure 2 the dashed line represents the average effect, and the plotted line shows what happened to the effect when additional variance is taken into consideration. As can be seen in Figure 2a, the indirect effect only disappeared (i.e., crosses the *x*-axis) when additional variance was very high, compared to the lower variance required for the loss of the direct effect (Figure 2b).[Fig-anchor fig2]

A similar, reverse, story was observed when autobiographical memory was used as the mediator between scene construction and future thinking. The autobiographical memory indirect sensitivity rapidly crossed the *x*-axis (ρ = .1, Figure 2c) in comparison to the much higher sensitivity of the direct scene construction to future thinking relationship (ρ = −.95, Figure 2d). Overall, therefore, the effect of scene construction (both as a mediator and directly) was considerably more robust than autobiographical memory, lending additional support to our mediation results. In summary, these first mediation analyses showed that scene construction could explain the relationship between autobiographical memory and future thinking. On the other hand, autobiographical memory could not explain the scene construction-future thinking relationship.

**Figure 3 fig3:**
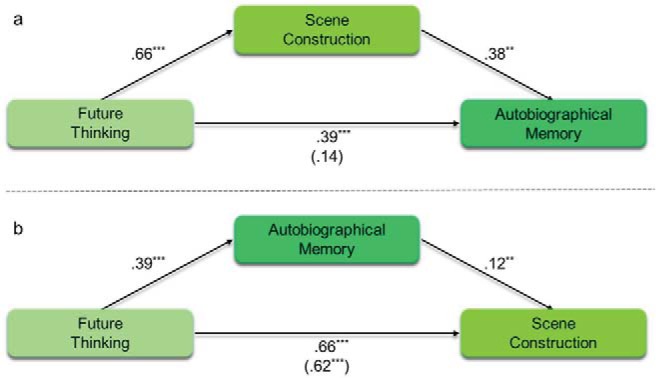
Mediation analyses of the scene component variables, when future thinking is the independent variable. (a) Future thinking to autobiographical memory, mediated by scene construction. (b) Future thinking to scene construction, mediated by autobiographical memory. The numbers in brackets show the effect of the independent variable on the dependent when the mediation variable was also taken into consideration. ** *p* < .01. *** *p* < .001.

Next, we investigated the relationships within the scene component when future thinking was included as the independent instead of the dependent variable (Table 5, Figure 3). As would be expected, future thinking was associated with both autobiographical memory (β = .39, *p* < .001) and scene construction (β = .66, *p* < .001). This shows that mediation was possible by both autobiographical memory and scene construction (full regression details are provided in Supplemental Table S9 in the online supplementary material). However, as before, whereas the relationship between future thinking and autobiographical memory was fully mediated by scene construction (Table 5a and Figure 3a), the relationship between future thinking and scene construction was only partially mediated by autobiographical memory (Table 5b and Figure 3b). That is, although scene construction could fully explain the relationship between future thinking and autobiographical memory, future thinking was still associated with scene construction even with the additional presence of autobiographical memory.[Table-anchor tbl5][Fig-anchor fig3]

**Figure 4 fig4:**
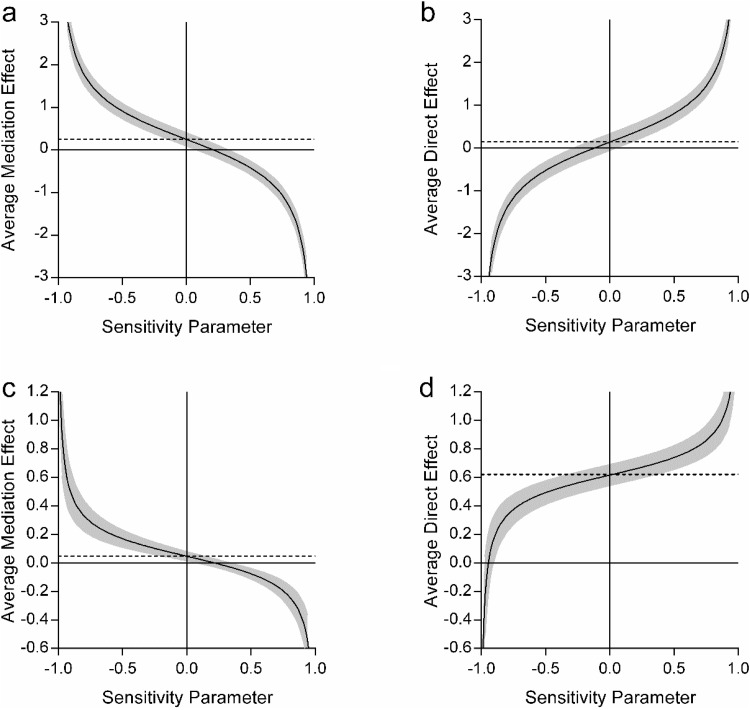
Sensitivity analyses for the indirect and direct effects of the mediation analyses of the scene component variables when future thinking was the independent variable. (a) Sensitivity of the indirect effect of scene construction on the relationship between future thinking and autobiographical memory. (b) Sensitivity of the direct effect between future thinking and autobiographical memory, when scene construction was taken into consideration. (c) Sensitivity of the indirect effect of autobiographical memory on the relationship between future thinking and scene construction. (d) Sensitivity of the direct effect between future thinking and scene construction, when autobiographical memory was taken into consideration. The dashed line shows the average effect when additional error is assumed to be 0. The plotted line shows the variation in the effect when the additional error is varied between −1 and 1 (with 95% confidence intervals). The more robust the effect, the greater the variance that was required to reduce the effect to 0 (i.e., to cross the *x*-axis).

Looking at the sensitivity analyses, the indirect effect of scene construction on the future thinking-autobiographical memory relationship was small but robust in comparison to the nonsignificant direct effect of future thinking (ρ = .2 vs. ρ = −.1; Figures 4a and 4b respectively). This supports the mediating role of scene construction on the relationship between future thinking and autobiographical memory. When comparing the sensitivity values for the mediation of autobiographical memory on the future thinking–scene construction relationship, the direct relationship between future thinking and scene construction was much more robust (ρ = −.95, Figure 4d) than the indirect effect of autobiographical memory (ρ = .2, Figure 4c). This highlights that although autobiographical memory may have been contributing something additional to the future thinking–scene construction relationship, it was to a much lesser extent than that of future thinking itself.[Fig-anchor fig4]

Finally, for completeness, we also examined whether future thinking mediated the relationship between scene construction and autobiographical memory. Although theoretical accounts emphasize either scene construction or autobiographical memory as the underlying cognitive process of future thinking, the reverse remains possible. However, as can be seen in Table 5a and Figure 3a there was no direct effect between future thinking and autobiographical memory with the inclusion of scene construction as the mediator. This, therefore, suggests that future thinking cannot explain the relationship between scene construction and autobiographical memory.

Overall, following a systematic examination of the different configurations of scene construction, autobiographical memory, and future thinking, we observed a consistent mediation by scene construction in the various combinations of the relationships between our tasks of primary interest. On the other hand, autobiographical memory seemed to have only limited input. Furthermore, future thinking also failed to explain the relationship between autobiographical memory and scene construction.

In summary, we aimed to assess the underlying psychological process of the scene component. We predicted that the process of scene construction would best explain the relationships between the three tasks. In line with our prediction, scene construction fully mediated the relationship from autobiographical memory to future thinking and from future thinking to autobiographical memory. Autobiographical memory recall, on the other hand, did not contribute to the relationship from scene construction to future thinking, and only partially mediated the effect of future thinking on scene construction. It seems, therefore, that a key process underpinning the scene component is indeed related to the mental construction of scene imagery.

### How Does the Scene Component Relate to Navigation?

The scene component only contained three of our tasks of primary interest. The fourth, navigation, aligned instead with the spatial component. Nevertheless, navigation has long been associated with hippocampal function (Maguire et al., 2000; O’Keefe & Nadel, 1978) and more recently with scene construction (Maguire & Mullally, 2013). Consequently, we also tested whether there was any kind of relationship between the tasks of the scene component and navigation.

To do this, we performed mediation analyses involving scene construction, autobiographical memory, future thinking, and navigation. We aimed to establish whether there was an underlying link between the scene component variables and navigation, predicting that there would be, and that this would be scene construction and not autobiographical memory. As such, we first investigated whether scene construction mediated the relationship between autobiographical memory and navigation. This was then followed by examination of whether autobiographical memory mediated the relationship between scene construction and navigation.

**Figure 5 fig5:**
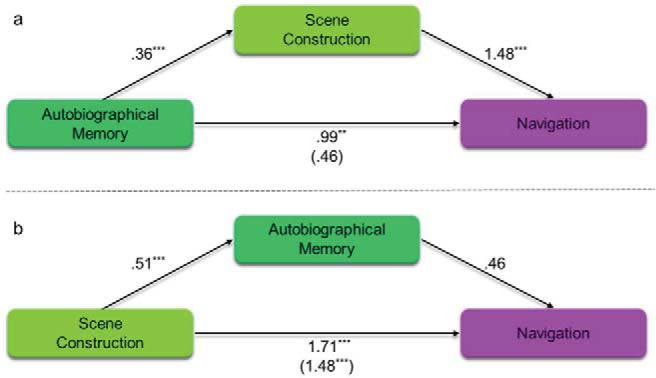
Mediation analyses of the scene construction, autobiographical memory and navigation relationships. (a) Autobiographical memory to navigation, mediated by scene construction. (b) Scene construction to navigation, mediated by autobiographical memory. The numbers in brackets show the effect of the independent variable on the dependent when the mediation variable was also taken into account. ** *p* < .01. *** *p* < .001.

The results of the mediation analyses are shown in Table 6 and Figure 5 (see also Supplemental Table S10 in the online supplementary materials for the full break down of the regression analyses). Figure 5a shows the relationship between autobiographical memory and navigation with scene construction as the mediator. First, we observed that autobiographical memory was related to both scene construction (β = .36, *p* < .001) and navigation (β = .99, *p* = .003). This confirmed that mediation by scene construction was possible. Then, importantly, we found that with scene construction included as the mediator, autobiographical memory was no longer related to navigation (β = .46, *p* = .2), while scene construction was (β = 1.48, *p* < .001). Mediation analysis revealed this to be a significant indirect effect of scene construction, with a nonsignificant direct effect of autobiographical memory (Table 6a and Figure 5a). This suggested that scene construction fully explained the relationship between autobiographical memory and navigation.[Table-anchor tbl6][Fig-anchor fig5]

In contrast, Figure 5b shows the relationship between scene construction and navigation mediated by autobiographical memory. Again, we found that scene construction was related to both autobiographical memory (β = .51, *p* < .001) and navigation (β = 1.71, *p* < .001). As such, mediation by autobiographical memory was possible. However, when autobiographical memory was included as the mediator, no relationship was found between autobiographical memory and navigation (β = .46, *p* = .2). Notably, the direct effect between scene construction and navigation remained significant (β = 1.48, *p* < .001). Mediation analyses confirmed a significant direct effect in the absence of an indirect effect (Table 6b). This, therefore, suggests that autobiographical memory had no influence on the relationship between scene construction and navigation.

**Figure 6 fig6:**
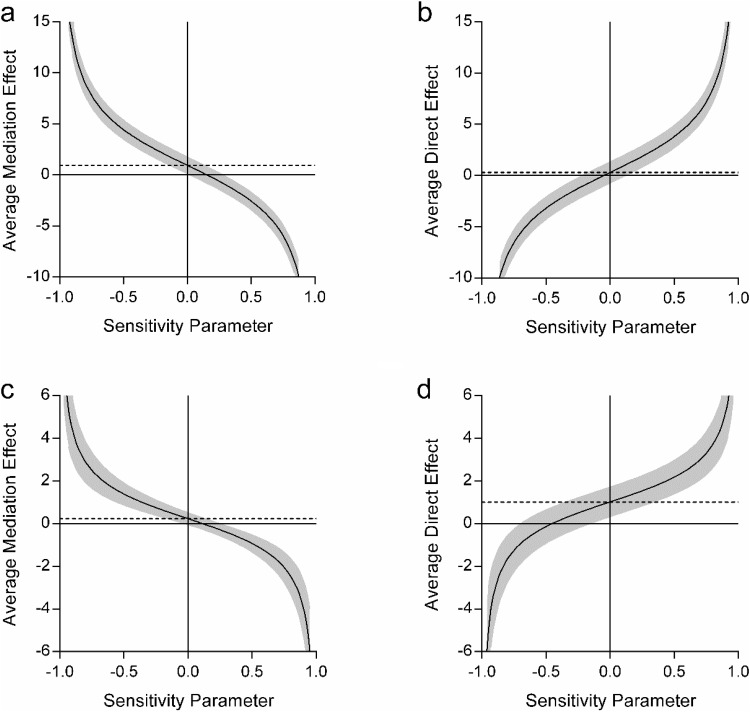
Sensitivity analyses for the indirect and direct effects of the mediation analyses of the scene construction, autobiographical memory and navigation relationships. (a) Sensitivity of the indirect effect of scene construction on the relationship between autobiographical memory and navigation. (b) Sensitivity of the direct effect between autobiographical memory and navigation, when scene construction was taken into consideration. (c) Sensitivity of the indirect effect of autobiographical memory on the relationship between scene construction and navigation. (d) Sensitivity of the direct effect between scene construction and navigation, when autobiographical memory was taken into consideration. The dashed line shows the average effect when additional error is assumed to be 0. The plotted line shows the variation in the effect when the additional error is varied between −1 and 1 (with 95% confidence intervals). The more robust the effect, the greater the variance that was required to reduce the effect to 0 (i.e., to cross the *x*-axis).

Sensitivity analyses supported both sets of findings, suggesting more robust effects of scene construction than autobiographical memory. This was apparent both when scene construction was mediating the relationship of autobiographical memory and navigation, and for the direct relationship between scene construction and navigation (Table 6 and Figure 6). Overall, these results suggest that scene construction may underpin the relationship between autobiographical memory and navigation.[Fig-anchor fig6]

**Figure 7 fig7:**
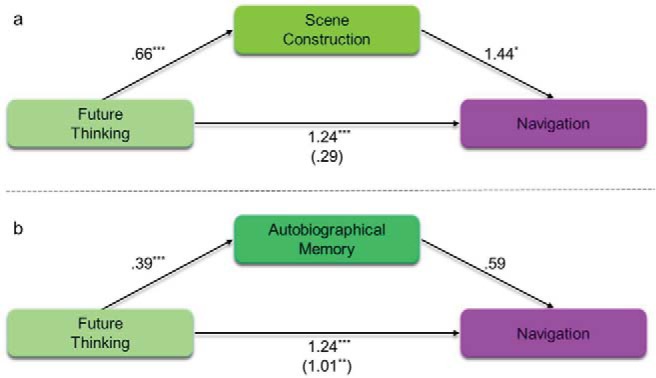
Mediation analyses of the future thinking to navigation relationship with scene construction or autobiographical memory as the mediating variable. (a) Future thinking to navigation, mediated by scene construction. (b) Future thinking to navigation, mediated by autobiographical memory. The numbers in brackets show the effect of the independent variable on the dependent when the mediation variable was also taken into consideration. * *p* < .05. ** *p* < .01. *** *p* < .001.

We next investigated whether scene construction would also mediate the future thinking to navigation relationship. Once again, we compared this to the mediating ability of autobiographical memory. The results of the mediation analyses are shown in Table 7 and Figure 7 (see Supplemental Table S11 in the online supplementary materials for the individual regressions). Figure 7a shows the relationship between future thinking and navigation, mediated by scene construction. Future thinking was related to both navigation (β = 1.24, *p* < .001) and scene construction (β = .66, *p* < .001). This confirmed that mediation by scene construction was possible. With the inclusion of scene construction as the mediator, future thinking was no longer related to navigation (β = .29, *p* = .58), while scene construction was (β = 1.44, *p* = .022). Mediation analysis identified a significant indirect effect of scene construction, with no direct effect of future thinking (Table 7a). This, therefore, suggests that scene construction fully mediated the relationship between future thinking and navigation, in addition to mediating the autobiographical memory to navigation relationship reported above.[Table-anchor tbl7][Fig-anchor fig7]

On the other hand, Figure 7b shows the relationship between future thinking and navigation, mediated by autobiographical memory. Future thinking was again found to be related to both autobiographical memory (β = .39, *p* < .001) and navigation (β = 1.24, *p* < .001). This confirmed that mediation by autobiographical memory was possible. However, including autobiographical memory as the mediating variable had limited effect; future thinking remained associated with navigation (β = 1.01, *p* = .0045) and there was no relationship between autobiographical memory and navigation (β = .59, *p* = .093). Mediation analysis confirmed the absence of an indirect effect of autobiographical memory and the presence of a significant direct effect from future thinking to navigation (Table 7b).

**Figure 8 fig8:**
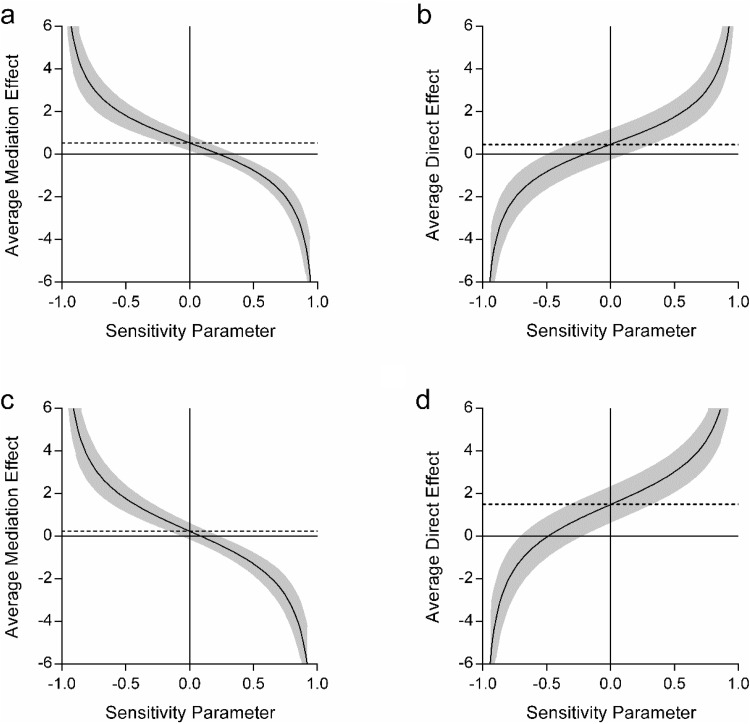
Sensitivity analyses for the indirect and direct effects of the mediation analyses of the future thinking to navigation relationship with scene construction or autobiographical memory as the mediating variable. (a) Sensitivity of the indirect effect of scene construction on the relationship between future thinking and navigation. (b) Sensitivity of the direct effect between future thinking and navigation, when scene construction was taken into consideration. (c) Sensitivity of the indirect effect of autobiographical memory on the relationship between future thinking and navigation. (d) Sensitivity of the direct effect between future thinking and navigation, when autobiographical memory was taken into consideration. The dashed line shows the average effect when additional error is assumed to be 0. The plotted line shows the variation in the effect when the additional error is varied between −1 and 1 (with 95% confidence intervals). The more robust the effect, the greater the variance that was required to reduce the effect to 0 (i.e., to cross the *x*-axis).

As before, sensitivity analyses were performed to test for the robustness of the effects. These showed, first, a more robust indirect effect of scene construction (ρ = .15, Figure 8a) than the direct relationship between future thinking and navigation (ρ = −.05, Figure 8b). Second, a more robust direct effect of future thinking on navigation (ρ = −.45, Figure 8d) in comparison to the indirect effect of autobiographical memory (ρ = .1, Figure 8c). This supports the mediation analyses.[Fig-anchor fig8]

We do, however, note that here we have two possible mediators for the future thinking navigation relationship. In addition, the finding of a significant indirect effect of scene construction in comparison to the absence of an indirect effect of autobiographical memory does not necessarily confirm that scene construction is more important than autobiographical memory. We therefore performed an additional analysis with both scene construction and autobiographical memory included as potential mediators on the future thinking navigation relationship at the same time. We found a significant indirect effect of scene construction (β = .84; 95% CI [.015, 1.67], *p* = .046) in the absence of an indirect effect of autobiographical memory (β = .17; 95% CI [−.10, .45], *p* = .22) and no direct relationship between future thinking and navigation (β = .23; 95% CI [−.79, 1.26], *p* = .66). This, therefore, supports our previous analyses in demonstrating the importance of scene construction, and the absence of the influence of autobiographical memory, in relating the scene component to navigation.

### Does Scene Construction Retain Influence on Navigation When Spatial Processing Is Taken Into Account?

The results so far suggest that the process of scene construction may underpin the relationship between our main tasks of primary interest (i.e., scene construction, autobiographical memory, future thinking and navigation). However, it is important to acknowledge that in our initial PCA, navigation loaded on the spatial component and not the scene component. This tells us that although scene processing may have some relationship with navigation (as shown by the analyses above), navigation is still closely associated with spatial processing. Consequently, this raises the question of whether scene construction only plays a role in the relationship between the scene component tasks and navigation in the absence of spatial processing.

To investigate this, we took a similar mediation approach as before, now using the spatial and scene components of the PCA. As such, we asked whether the tasks of the scene component would mediate the relationship between the tasks of the spatial component and navigation. We did this in two ways. First, we examined the effects of including all three tasks of the scene component together as a combined mediator variable, to balance the inclusion of the combined spatial component tasks as the independent variable. Second, we placed scene construction, autobiographical memory and future thinking in turn as separate mediator variables to see if the three tasks of the scene component had differing mediating effects on the spatial component to navigation relationship.

Latent variables were used to represent the scene and spatial components. The latent variables were comprised of the tasks that loaded singularly onto the respective components. This allowed for assessment of only the pure elements of each component. For the spatial component this was the ROCF (delayed recall), the Paper Folding Test, the Object-Place Association Test, and the Brixton Spatial Anticipation Test. For the scene component, the tests were scene construction, autobiographical memory, and future thinking. To perform a mediation analysis using latent variables, a structural equation modeling (SEM) approach was taken. Aside from the inclusion of latent variables, however, the principles of the analysis remained the same as the mediation analyses reported above. The only exception being that sensitivity analyses can no longer be conducted; judgments are made in SEM on the goodness of model fit.

**Figure 9 fig9:**
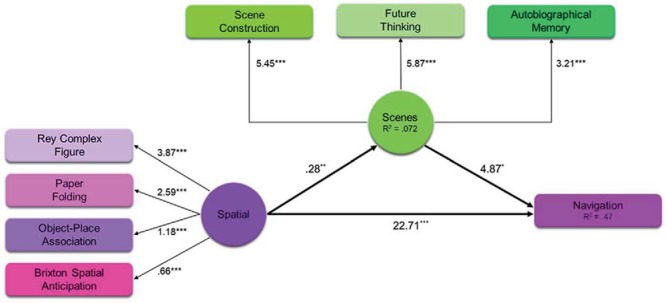
Structural equation model of the spatial component to navigation relationship mediated by the scene component. The darker arrows show the main paths of interest, the lighter arrows show the links between the individual observed variables and their related latent variable. The *R*^2^ values represent the proportion of variance explained by the main paths of interest (i.e., the dark arrows). Numerical values linked with a pathway represent unstandardized path coefficients. * *p* < .05. ** *p* < .01. *** *p* < .001.

Figure 9 shows the SEM of the relationship between the spatial component and navigation, mediated by the scene component (see also see Supplemental Table S12 in the online supplementary materials for full details of individual paths). The latent variables (spatial and scene PCA components) are shown in circles, the observed variables (the cognitive tasks) in rectangles. The numerical values represent the unstandardized coefficients of the path in question. Overall model fit was good, in line with published recommendations (Hu & Bentler, 1999), χ^2^(18) = 20.70, *p* = .30; comparative fit index (CFI) = .99; Tucker-Lewis index (TLI) = .99; root mean square error of approximation (RMSEA) = .026, 90% CI [0, .068]; standardized root mean square residual (SRMR) = .035. As would be expected, the ROCF, the Paper Folding Test, the Object-Place Association Test, and the Brixton Spatial Anticipation Test all loaded significantly onto the spatial latent variable (Beta coefficients respectively of 3.87, *p* < .001; 2.59, *p* < .001; 1.18, *p* < .001; .66, *p* < .001). In addition, scene construction, future thinking, and autobiographical memory all loaded significantly onto the scene latent variable (Beta coefficients, respectively, of 5.45, *p* < .001; 5.87, *p* < .001; 3.21, *p* < .001). Of key relevance to our question of interest, the spatial component was associated with the scene component (β = .28, *p* = .002), and both the spatial and scene components were associated with navigation (β = 22.71, *p* < .001; β = 4.87, *p* = .03, respectively). This indicates that the scene component partially mediated the relationship between the spatial component and navigation. This is supported by a mediation analysis finding a significant indirect effect of the scene component (β = 1.35; 95% CI [.093, 2.62], *p* = .035). Unsurprisingly, the spatial component remained associated with navigation even with the introduction of the Scene component.[Fig-anchor fig9]

Hence, we see a partial mediation by the scene component in comparison to the full mediations observed earlier. Overall, this suggests that scene processing had an influence on navigation even when the spatial component was taken into account.

**Figure 10 fig10:**
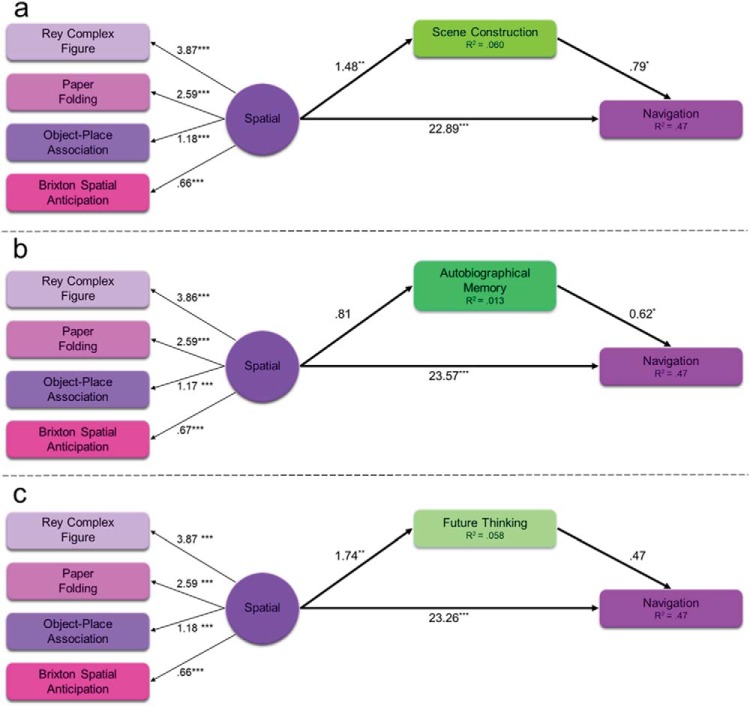
Structural equation models of the spatial component to navigation relationship mediated by scene construction, autobiographical memory or future thinking. The darker arrows show the main paths of interest, the lighter arrows show the links between the individual observed variables and the latent variable (spatial). The *R*^2^ values represent the proportion of variance explained by the main paths of interest (i.e., the dark arrows). Numerical values linked with a pathway represent unstandardized path coefficients. * *p* < .05. ** *p* < .01. *** *p* < .001.

As the scene component was made up of three variables, we next tested whether the partial mediation by the scene component on the spatial component to navigation relationship was specifically due to scene construction or could also be explained by autobiographical memory or future thinking. We therefore repeated the SEM three more times, replacing the scene component with each individual task in turn. Figure 10 shows the results of the three SEMs using scene construction, autobiographical memory, or future thinking as the mediator on the spatial component to navigation relationship. As before, all models showed acceptable fit—scene construction mediation: χ^2^(8) = 14.84, *p* = .062; CFI = .97; TLI = .94; RMSEA = .063 (90% CI [0, .11]); SRMR = .038; autobiographical memory mediation: χ^2^(8) = 15.04, *p* = .058; CFI = .97; TLI = .94; RMSEA = .064 (90% CI [0, .11]); SRMR = .038; and future thinking mediation: χ^2^(8) = 15.43, *p* = .051; CFI = .97; TLI = .94; RMSEA = .065 (90% CI [0, .11]); SRMR = .039.[Fig-anchor fig10]

Notably, the patterns of mediation differed in each model. As can be seen in Figure 10a (see also Supplemental Table S13 in the online supplementary materials), when scene construction was used as the mediator, an indirect effect was observed. The spatial component was associated with scene construction (β = 1.48, *p* = .002) and both the spatial component and scene construction were associated with navigation (β = 22.89, *p* < .001; β = .79, *p* = .026, respectively). This indicates that, just like the overall scene component, scene construction partially mediated the relationship between the Spatial component and navigation. This was supported by a mediation analysis finding a significant indirect effect of scene construction (β = 1.17; 95% CI [.079, 2.26], *p* = .036).

On the other hand, Figure 10b (see also Supplemental Table S14 in the online supplementary materials), shows the effect of using autobiographical memory as the mediator. Whereas the spatial component continued to be associated with navigation (β = 23.57, *p* < .001), the spatial component was not associated with autobiographical memory (β = .81, *p* = .17). As such, although autobiographical memory itself was related to navigation (β = .62, *p* = .031), as there was no relationship between the spatial component and autobiographical memory; these effects were nonmediating. This was supported by the mediation analysis finding no indirect effect of autobiographical memory (β = .50; 95% CI [−.23, 1.24], *p* = .18).

Finally, Figure 10c (see also Supplemental Table S15 in the online supplementary materials) shows the indirect effect of future thinking. Here, the spatial component was associated with both future thinking (β = 1.74, *p* = .003) and navigation (β = 23.26, *p* < .001). However, there was no relationship between future thinking and navigation when the spatial component was taken into consideration (β = .47, *p* = .12). This suggests that future thinking had no indirect effect on the spatial component to navigation relationship. This was supported by the mediation analysis (β = .81; 95% CI [−.20, 1.83], *p* = .12).

Overall, therefore, we found that scene construction played a role in the relationship between spatial processing and navigation. This is observed by the indirect effects of both the overarching scene component, and more specifically when just using scene construction. On the other hand, neither autobiographical memory nor future thinking mediated the spatial component to navigation relationship. To that end, even in the presence of other highly associated spatial tasks, scene construction continued to be a key process involved in navigation.

## Discussion

Autobiographical memory, future thinking, spatial navigation and the imagination of scene imagery are critical cognitive functions that are typically regarded as being related, primarily because they are all hippocampal-dependent. Until now, direct evidence for their interrelatedness has been lacking, as has an understanding of why they might be related. There were four main findings from the current study that spoke to these issues. First, using a PCA, we found that, in the presence of other cognitive tasks, scene construction, autobiographical memory, and future thinking all loaded onto the same component, confirming a strong relationship between these variables. Navigation on the other hand, loaded more strongly with spatial tasks. Second, we showed that scene construction fully mediated the relationship between autobiographical memory and future thinking, while autobiographical memory did not mediate between scene construction and future thinking, nor did future thinking mediate between scene construction and autobiographical memory. Third, we found that scene construction fully mediated the relationships between future thinking and autobiographical memory with navigation, whereas autobiographical memory did not mediate the relationships between future thinking and scene construction with navigation. Finally, we observed a partial mediation by scene construction on the relationship between the spatial tasks and navigation, compared to no mediation of autobiographical memory or future thinking. Overall, our results suggest that scene construction may be a significant cognitive process underlying the relationships between these different functions that are each associated with the hippocampus.

The crucial role of visual imagery is well documented across multiple cognitive domains, including autobiographical memory, future thinking, and navigation (Andrews-Hanna, Saxe, & Yarkoni, 2014; Greenberg & Knowlton, 2014; Kraemer et al., 2017). Why might scene imagery in particular be at the heart of these important cognitive functions? One reason is that scene imagery allows us to build models of the world that mirror our moment-by-moment perception. Scenes are also a highly efficient means of packaging information and, as such, are an economical use of cognitive resources (e.g., Konkle, Brady, Alvarez, & Oliva, 2010). Through the construction of a visual scene we can incorporate event details of episodic memories and future events, or route details when navigating, allowing them to be played out in a coherent and naturalistic manner (Maguire & Mullally, 2013; see also Clark & Maguire, 2016).

Revealing the influence of scene construction over autobiographical memory may seem to be in contrast to the decades of work that has strongly associated the hippocampus and autobiographical memory (Cabeza & St. Jacques, 2007; Squire, 1992; Svoboda et al., 2006). We do not deny or diminish this relationship. However, in addition to autobiographical memory, scene construction and thinking about the future have also been associated with the hippocampus (Hassabis, Kumaran, Vann, et al., 2007; Schacter et al., 2012), and there are substantial overlaps in the behavioral correlates of autobiographical memory, scene construction and future thinking (D’Argembeau & Van der Linden, 2004; de Vito et al., 2012; Robin & Moscovitch, 2014). We suggest that our results allow us to start specifying more precisely why these similar, but different, cognitive processes are associated with the hippocampus. In short, our findings point toward scene construction being a common process underlying autobiographical memory and future thinking (Maguire & Mullally, 2013; Zeidman & Maguire, 2016) rather than autobiographical memory being the common component (Addis et al., 2007; Schacter et al., 2012).

It is interesting to note that the PCA loaded navigation with spatial tasks, and not with scene construction, autobiographical memory, and future thinking. Navigation also had the smallest effect sizes in terms of the regressions among the primary tasks of interest. Why this is the case will be an interesting topic for future work. For now, we have two speculations. First, imagery comes in multiple forms. A popular distinction is between analytical imagery, reliant upon schematic images, compared to vivid and colorful images of specific scenes and objects (e.g., Kozhevnikov, Kosslyn, & Shephard, 2005). It could be argued that navigation is more like the former, whereas scene construction, autobiographical memory, and future thinking are more similar to the latter. A detailed analysis of the types of imagery being used to perform these tasks may be useful in exploring this further. Second, the distinction between navigation and the other tasks may be because they rely on different hippocampal subregions. Navigation is typically associated with the posterior hippocampus (Maguire et al., 2000), whereas scene construction, autobiographical memory, and future thinking are more often associated with the anterior hippocampus (Dalton & Maguire, 2017; Maguire & Mullally, 2013; Zeidman & Maguire, 2016). Understanding the specialization of different regions of the hippocampus will also be an important topic for future work.

Although the reduced associations with navigation advocate caution in making generalizations from navigation studies to, for example, autobiographical memory, we nevertheless still found that scene construction partially mediated the relationship between the spatial tasks and navigation. Thus, even with navigation being more strongly associated with spatial tasks, the involvement of scene processing remained prominent, whereas, importantly, neither autobiographical memory nor future thinking mediated this relationship.

Here, our main interest was in scene construction, autobiographical memory, future thinking, and navigation. As such, the numerous other tasks that were included in the initial PCA are not reported on in detail. However, we make several brief observations in relation to these tests. It is notable that recall and recognition tasks loaded onto separate components, as did episodic and semantic memory tasks. There is still debate in the literature about whether all of these tasks are hippocampal-dependent (Smith et al., 2014; Squire, 1992) or whether only recall and episodic memory tasks require the hippocampus (Eichenbaum, Yonelinas, & Ranganath, 2007). Although we do not assess this in detail, our findings are more concordant with this latter perspective.

It is also the case that the recall tasks loaded onto components that were different from the scene component onto which our primary tasks of interest loaded. If the hippocampus is involved in supporting memory recall tasks and also scene construction, autobiographical memory and future thinking, why did they all not cluster onto one factor? The data suggest that the standardized tests, in particular, clustered according to the modality in which a test was presented. That is, all the verbal recall tasks loaded together, and the visual recall tasks loaded on the spatial component. This does not mean that these tasks are unrelated to our primary tasks of interest, but rather that modality exerted a significant influence.

One question that is often asked of the scene construction theory, is why does hippocampal damage result in verbal memory deficits, for example, in word paired associates tasks, if visuospatial scenes are of particular relevance for hippocampal function? We have previously suggested that some verbal tasks may in fact engage scene imagery (e.g., imagining the two objects in a word pair together in a scene; Clark & Maguire, 2016; Maguire & Mullally, 2013), and that this could explain their dependence on the hippocampus. Recent work using functional neuroimaging lends credence to this idea by finding that high imagery concrete word pairs evoked hippocampal activity due to the use of scene imagery, whereas low imagery abstract word pairs did not (Clark et al., 2018). Another way to test this in the future would be to interrogate the explicit strategies that people use to perform different verbal recall tasks. This would enable us to ascertain if scene imagery is involved more generally in verbal tasks, and indeed whether the use of such imagery confers a performance advantage.

We note that the scene component of the PCA contained tasks that were scored from open ended verbal descriptions. As such, verbal task demands—be that narrative style, verbal ability, and so forth—or similarities in scoring across the tasks could be candidate processes linking scene construction, autobiographical memory and future thinking. However, if this was the case, we would have expected a different pattern of results to emerge. First, autobiographical memory external details should have loaded onto the scene component, and it did not. Second, the loading of the scene description task should have been stronger, more in line with the loadings of scene construction, future thinking and autobiographical memory, but it was not. Finally, future thinking should have mediated the relationship between autobiographical memory and scene construction and the relationship between the spatial component and navigation, and yet it did not. Instead, we observed that external details loaded onto the semantic memory component, that the scene description task loaded most strongly on the perception component and that there was only a mediating effect of scene construction.

In addition, to further examine the potential involvement of verbal processing, we also ran a series of control mediation analyses looking at the effects of the verbal memory component (as a proxy for verbal ability) on the tasks of the scene component (see Supplemental Figure S2 in the online supplementary materials). We found that the influence of the verbal memory component was either fully or partially mediated in all the models. This suggests that the relationships between scene construction, autobiographical memory and future thinking we reported above cannot simply be explained by verbal ability.

A related potential criticism is that we tested the influence of memory on the relationship between scene construction, autobiographical memory and future thinking using a memory task that relied on verbal output. To address this issue, we also examined the relationships between scene construction, autobiographical memory and future thinking with a nonverbal memory task (the delayed recall of the ROCF) as the mediator and found no influence on any of the relationships (see Supplemental Tables S15 and >S16 in the online supplementary materials). This lends further support to the idea that scene imagery rather than memory may be a key feature underlying the relationships between scene construction, autobiographical memory, and future thinking.

Finally, we also observed the surprising finding in the PCA analysis that the Brixton Spatial Anticipation Test, Matrix Reasoning, and the Symbol Span test loaded most strongly on the spatial component. This was unexpected because these tasks are typically thought to tax executive functioning and general intellectual ability (e.g., Wechsler, 2008, 2009). Studies using these standardized tasks should perhaps bear this in mind, as our data suggest that individual differences in spatial processing could affect performance on these tasks.

Here we have alluded to the function of the hippocampus without measuring the hippocampus itself. We feel confident in doing so because of the many previous findings associating the hippocampus with scene construction, autobiographical memory, future thinking and navigation. Moreover, the issue of central interest here—to understand the cognitive processes involved in these tasks—is not reliant upon direct hippocampal measurement. However, an important next step will undoubtedly be to directly relate the process of scene construction with structural and functional measurements of the hippocampus.

We also acknowledge that scene construction, autobiographical memory, future thinking, and navigation have each been associated with brain regions outside of the hippocampus including (but not limited to) parahippocampal, retrosplenial, posterior cingulate, parietal, and medial prefrontal cortices (e.g., Hassabis, Kumaran, & Maguire, 2007; Schacter et al., 2012; Stawarczyk & D’Argembeau, 2015). For example, lesions to the parietal cortex impair the subjective experience associated with autobiographical memory (Ciaramelli et al., 2017; Simons, Peers, Mazuz, Berryhill, & Olson, 2010) and posterior parietal damage has been linked to a reduction in scene construction ability (Ramanan et al., 2018). A variety of extrahippocampal brain regions have been implicated in supporting navigation, and a reliance upon nonhippocampal regions for navigation has been suggested to increase with age (Moffat, Elkins, & Resnick, 2006; Zhong & Moffat, 2018). An important future step will, therefore, be to understand the interactions, both structural and functional, between the hippocampus and these other regions, and their relationships with individual differences in task performance.

We also note that it is unlikely the hippocampus supports only one fundamental process. A number of studies have associated different hippocampal subregions with distinct cognitive processes (e.g., Dalton, Zeidman, McCormick, & Maguire, 2018; Dimsdale-Zucker, Ritchey, Ekstrom, Yonelinas, & Ranganath, 2018; Hodgetts et al., 2017; Zeidman, Lutti, & Maguire, 2015). The results from the current study suggest that the construction of scene imagery seems to play an influential role in autobiographical memory, future thinking and, to some extent, navigation, and consequently, scene construction may be at least one process performed by the hippocampus.

In conclusion, we are not alone in suggesting that the hippocampus is more than just a memory device (O’Keefe & Nadel, 1978; Shohamy & Turk-Browne, 2013; Tulving, 2002; Verfaellie & Keane, 2017). However, here, a large sample of participants, numerous cognitive tests, and a wide variance in performance enabled us to provide novel evidence regarding the interrelations between tasks that have hitherto not been systematically examined. We found that the construction of scene imagery plays a particularly prominent role in several hippocampal-dependent tasks. This finding lays the groundwork for future studies that should directly examine the strategies and types of imagery people use to perform such tasks, and how this is realized by the hippocampus and its specific subregions.

## Context

The current study is part of a body of work investigating the relationships between a diversity of tasks that have individually been associated with a brain structure called the *hippocampus*, located deep in the brain’s temporal lobes. There is debate about the hippocampus’ contribution to these tasks which, on face value, appear to be distinct. Here, we highlight the importance of visual scene imagery for three tasks typically associated with the hippocampus—autobiographical memory, future thinking, and spatial navigation. Future work aims to build on these findings by conducting a detailed analysis of the explicit strategies deployed by participants to perform these tasks and whether scene imagery plays a role, and by examining how variations in task performance may be related to structural and functional measurements of the brain, including the hippocampus.

## Supplementary Material

10.1037/xge0000582.supp

## Figures and Tables

**Table 1 tbl1:** Means With 95% Confidence Intervals (CIs) for All Cognitive Tasks

Variable	Mean	Lower CI	Upper CI
Scene Construction Experiential Index (/60)	40.50	29.50	50.13
Autobiographical memory internal details (total number)	23.95	13.80	37.42
Autobiographical memory external details (total number)	5.35	1.40	11.24
Future Thinking Experiential Index (/60)	39.12	25.00	49.99
Navigation (/250)	143.46	88.90	201.50
Full Scale Intelligence Quotient^a^	102.75	92.04	114.35
Matrix Reasoning scaled score (/19)	12.53	8.00	17.00
Brixton Spatial Anticipation Test scaled score (/10)	7.87	5.00	10.00
F-A-S Verbal Fluency (total number of words)	49.09	30.90	69.00
Digit Span scaled score (/37—sum of backwards and forwards)	22.08	14.00	34.00
Symbol Span scaled score (/19)	9.35	6.00	13.00
Rey-Osterrieth Complex Figure delayed recall (/36)	22.28	12.45	31.00
Object-Place Association Test (/6)	2.31	.00	6.00
Rey Auditory Verbal Learning Test delayed recall (/15)	12.92	8.90	15.00
Logical Memory delayed recall scaled score (/19)	12.58	8.00	17.00
WMS Verbal Paired Associates delayed recall (/14)	13.38	10.00	14.00
Concrete Verbal Paired Associates delayed recall (/14)	12.94	8.00	14.00
Abstract Verbal Paired Associates delayed recall (/14)	7.03	1.00	13.10
Warrington Recognition Memory Test for Words scaled score (/15)	12.75	7.00	14.00
Warrington Recognition Memory Test for Faces scaled score (/18)	11.00	4.00	16.00
Warrington Recognition Memory Test for Scenes raw score (/50)	43.35	35.00	49.00
Dead or Alive Task proportion correct (%)	81.32	66.12	94.52
Paper Folding Test (/20)	13.14	6.00	19.00
Scene description (total number of details)	24.88	15.90	37.10
Boundary extension proportion of “closer” responses (%)	42.26	8.33	79.17
*Note*. WMS = Wechsler Memory Scale; CI = confidence interval. Task order is for display purposes only.			
^a^ Estimated from the Test of Premorbid Functioning.			

**Table 2 tbl2:** Proportion of Variance Explained by Each Principal Component Analysis Component

PCA component	Variance explained
Total	59.24%
C1. Spatial processing	12.44%
C2. Verbal memory	10.64%
C3. General IQ/Executive function	9.85%
C4. Scenes	9.23%
C5. Recognition memory	6.81%
C6. Semantic memory	5.28%
C7. Perception	5.01%

**Table 3 tbl3:** Details of the Principal Component Analysis With Varimax Rotation of the Cognitive Tasks

Cognitive task	Spatial	Verbal	IQ/executive function	Scenes	Recognition memory	Semantic memory	Perception
Rey-Osterrieth Complex Figure delayed recall	.72						
Paper Folding Test	.72						
Navigation	.66						
Object-Place Association Test	.65						
Brixton Spatial Anticipation Test	.42						
Warrington Recognition Memory Test for Scenes	.54				.41		
Matrix Reasoning	.51		.49				
Symbol Span	.46		.46				
Rey Auditory Verbal Learning Test delayed recall		.74					
Concrete Verbal Paired Associates delayed recall		.67					
Logical Memory delayed recall		.66					
WMS Verbal Paired Associates delayed recall		.62					
Abstract Verbal Paired Associates delayed recall		.61	.46				
Digit Span			.74				
Full Scale Intelligence Quotient			.68				
F-A-S Verbal Fluency			.62				
Scene Construction Experiential Index				.87			
Future Thinking Experiential Index				.85			
Autobiographical Memory Internal Details				.62			
Scene Description				.37			.50
Warrington Recognition Memory Test for Words					.67		
Warrington Recognition Memory Test for Faces					.79		
Autobiographical Memory External Details						.69	
Dead or Alive Task						.62	
Boundary Extension							.84
*Note.* WMS = Wechsler Memory Scale. Task order is for display purposes only. Only values over .35 are reported for ease of viewing, full results are presented in Supplementary Materials Table S6 in the online supplemental material.							

**Table 4 tbl4:** Mediation Analyses of the Scene Component Variables When Future Thinking is the Dependent Variable

Effect	Beta [95% CI]	*p*	Sensitivity (ρ)
a			
Autobiographical memory to future thinking, mediated by scene construction			
Indirect effect	.32 [.23, .43]	<.001	.75
Direct effect	.06 [−.03, .15]	.17	−.2
Total	.39 [.26, .51]	<.001	n/a
b			
Scene construction to future thinking, mediated by autobiographical memory			
Indirect effect	.032 [−.014, .08]	.16	.1
Direct effect	.90 [80, 1.02]	<.001	−.95
Total	.94 [84, 1.04]	<.001	n/a
*Note.* CI = confidence interval.			

**Table 5 tbl5:** Mediation Analyses of the Scene Component Variables When Future Thinking is the Independent Variable

Effect	Beta [95% CI]	*p*	Sensitivity (ρ)
a			
Future thinking to autobiographical memory, mediated by scene construction			
Indirect effect	.25 [.10, .41]	<.001	.2
Direct effect	.14 [−.056, .32]	.16	−.1
Total	.39 [.27, .51]	<.001	n/a
b			
Future thinking to scene construction, mediated by autobiographical memory			
Indirect effect	.047 [.017, .08]	<.001	.2
Direct effect	.62 [.54, .69]	<.001	−.95
Total	.66 [.59, .73]	<.001	n/a
*Note.* CI = confidence interval.			

**Table 6 tbl6:** Mediation Analyses of the Scene construction, Autobiographical Memory and Navigation Relationships

Effect	Beta [95% CI]	*p*	Sensitivity
a			
Autobiographical memory to navigation, mediated by scene construction			
Indirect effect	.53 [.21, .90]	<.001	.25
Direct effect	.45 [−.25, 1.13]	.21	−.2
Total	.98 [.32, 1.64]	.003	n/a
b			
Scene construction to navigation, mediated by autobiographical memory			
Indirect effect	.23 [−.12, .61]	.20	.1
Direct effect	1.49 [.66, 2.33]	<.001	−.5
Total	1.72 [.99, 2.47]	<.001	n/a
*Note.* CI = confidence interval.			

**Table 7 tbl7:** Mediation Analyses of the Future Thinking to Navigation Relationship With Scene Construction or Autobiographical Memory as the Mediating Variable

Effect	Beta [95% CI]	*p*	Sensitivity
a			
Future thinking to navigation, mediated by scene construction			
Indirect effect	.94 [.11, 1.76]	.025	.15
Direct effect	.31 [−.73, 1.33]	.56	−.05
Total	1.25 [.60, 1.87]	<.001	n/a
b			
Future thinking to navigation, mediated by autobiographical memory			
Indirect effect	.23 [−.034, .54]	.088	.10
Direct effect	1.01 [.29, 1.70]	.0024	−.45
Total	1.25 [.60, 1.90]	<.001	n/a
*Note.* CI = confidence interval.
