# Supplementary material for: Identifying the Cognitive Processes Underpinning Hippocampal-Dependent Tasks
Source: J Exp Psychol Gen. 2019 Mar 4;148(11):1861–81. doi: 10.1037/xge0000582 (PMC6818684; doi:10.1037/xge0000582)
Supplement: Supplementary file 1 [file Clarkxge0000582.docx]

Clark et al. Supplementary Materials

Supplementary Methods

Table S1

*Double scoring of the scene construction test*

|  | | **Rating** | | | | |
| --- | --- | --- | --- | --- | --- | --- |
|  | **Spatial References** | | **Entities present** | **Sensory Descriptions** | **Thoughts/**  **Emotions/Actions** | **Quality ratings** |
| **For each individual scene** | | | | | | |
|  |  | |  |  |  |  |
| n = 308 | .90 | | .96 | .94 | .90 | .90 |
|  |  | |  |  |  |  |
| **For each individual participant (i.e. score is averaged across the seven scenes)** | | | | | | |
|  | | | | | | |
| n = 44 | .91 | | .99 | .97 | .91 | .93 |
|  |  | |  |  |  |  |

*Note.* Inter-class correlation coefficients from a two way random effect model looking for absolute agreement for each content score and for the quality ratings. Four experimenters scored the whole data set (n = 217 participants, 1519 individual scenes) with double scoring performed on 20% of the data (n = 44 participants, 308 scenes) proportionally for each original experimenter.

Table S2

*Double scoring of the Autobiographical Interview*

|  | | **Rating** | | | | | | |
| --- | --- | --- | --- | --- | --- | --- | --- | --- |
|  | **Internal Event** | | **Internal Place** | **Internal Time** | **Internal Perceptual** | **Internal Emotion** | **Internal Sum** | **External Sum** |
| **For each individual memory** | | | | | | |  |  |
|  |  | |  |  |  |  |  |  |
| n = 215 | .92 | | .85 | .94 | .92 | .86 | **.94** | **.84** |
|  |  | |  |  |  |  |  |  |
| **For each individual participant (i.e. score is averaged across the five memories)** | | | | | | | | |
|  | | | | | | |  |  |
| n = 43 | .95 | | .88 | .96 | .94 | .81 | **.97** | **.87** |
|  |  | |  |  |  |  |  |  |

*Note.* Inter-class correlation coefficients from a two way random effects model looking for absolute agreement for each score on the AI. Three experimenters scored the whole data set (n = 217 participants, 1085 individual memories) and double scoring was performed 20% of the data (n = 43 participants, 215 individual memories) proportionally for each original experimenter.

Table S3

*Double scoring of the future thinking test*

|  | | **Rating** | | | | |
| --- | --- | --- | --- | --- | --- | --- |
|  | **Spatial References** | | **Entities present** | **Sensory Descriptions** | **Thoughts/**  **Emotions/Actions** | **Quality ratings** |
| **For each individual scene** | | | | | | |
|  |  | |  |  |  |  |
| n = 132 | .90 | | .94 | .93 | .88 | .90 |
|  |  | |  |  |  |  |
| **For each individual participant (i.e. score is averaged across the three future scenes)** | | | | | | |
|  | | | | | | |
| n = 44 | .94 | | .95 | .96 | .88 | .92 |
|  |  | |  |  |  |  |

*Note.* Inter-class correlation coefficients from a two way random effects model looking for absolute agreement for each content score and for the quality ratings. Four experimenters scored the whole data set (n = 217 participants, 651 individual future scenes) with double scoring performed on 20% of the data (n = 44 participants, 132 future scenes) proportionally for each original experimenter.

Table S4

*Double scoring of the navigation sketch maps*

|  | **Rating** | | | | | | |
| --- | --- | --- | --- | --- | --- | --- | --- |
|  | | **Road Segments** | **Road Junctions** | **Number of Landmarks** | **Landmark Placement** | **Map Orientation** | **Map Categorisation** |
|  | |  |  |  |  |  |  |
| n = 42 | | .95 | .96 | .97 | .96 | .96 | .89 |
|  | |  |  |  |  |  |  |

*Note.* Inter-class correlation coefficients from a two way random effects model looking for absolute agreement for each score on the navigation sketch maps. Three experimenters scored the whole data set (n = 217) and double scoring was performed on 20% of the data (n = 42 participants) proportionally for each original experimenter.

Table S5

*Double scoring of the scene description task*

|  | | **Rating** | | | |
| --- | --- | --- | --- | --- | --- |
|  | **Spatial References** | | **Entities present** | **Sensory Descriptions** | **Thoughts/**  **Emotions/Actions** |
|  |  | |  |  |  |
| n = 43 | .88 | | .91 | .93 | .85 |
|  |  | |  |  |  |

*Note.* Inter-class correlation coefficients from a two way random effects model looking for absolute agreement for each content score. Three experimenters scored the whole data set (n = 217) with double scoring performed on 20% of the data (n = 43) proportionally for each original experimenter.

Supplementary Results

**How are the tasks interrelated? Additional results of the PCA**

The selection of the number of components in the solution of the PCA was performed using two methodologies. First, as detailed in the main body of the text, a minimum Eigen value set to one suggested a solution of seven components. However, as some of these components contained only two or three variables, we sought to validate the seven factor solution via a second methodology for component selection. This second approach used the “elbow” of the Scree plot of the Eigen values of each component. The Scree plot elbows detail where the explanatory power of the components begins to reduce, and thus become less useful for understanding the relationships in the data. However, it is not always clear where the elbow of the Scree plot is, and so potential solutions also have to be checked for interpretability. Choosing too few components may miss valid relationships in the data, while choosing too many can lead to the inclusion of components that account for only trivial amounts of variance and reduce the interpretability of more interesting components. As can be seen in Figure S1, the elbow of the Scree plot for the PCA could be placed in three locations, suggesting solutions of two, four or seven components. To identify the best solution, we looked at the interpretability of the three solutions.

When limiting the solution to two components, only 32% of the variance was explained. More importantly, two tasks – the Dead or Alive task and the boundary extension task – did not load on either component (highest loadings of .19 and .26 respectively). A two component solution was therefore deemed unsatisfactory and discounted.

When limiting to four components, the total variance explained remained low at only 45%. Importantly, once again, neither the Dead or Alive task nor the boundary extension task loaded onto any of the components (highest loadings of .26 and .27 respectively). A four component solution was therefore also deemed unsatisfactory and discounted.

However, as detailed in the main text, a solution of seven components not only explained 59% of the total variance but, crucially, loaded both the Dead or Alive and boundary extension tasks onto specific components of the PCA (loadings of .62 and .84 respectively). As such, both the minimum Eigen value methodology and the Scree plot methodology suggested the optimal solution of the PCA was that of seven components.


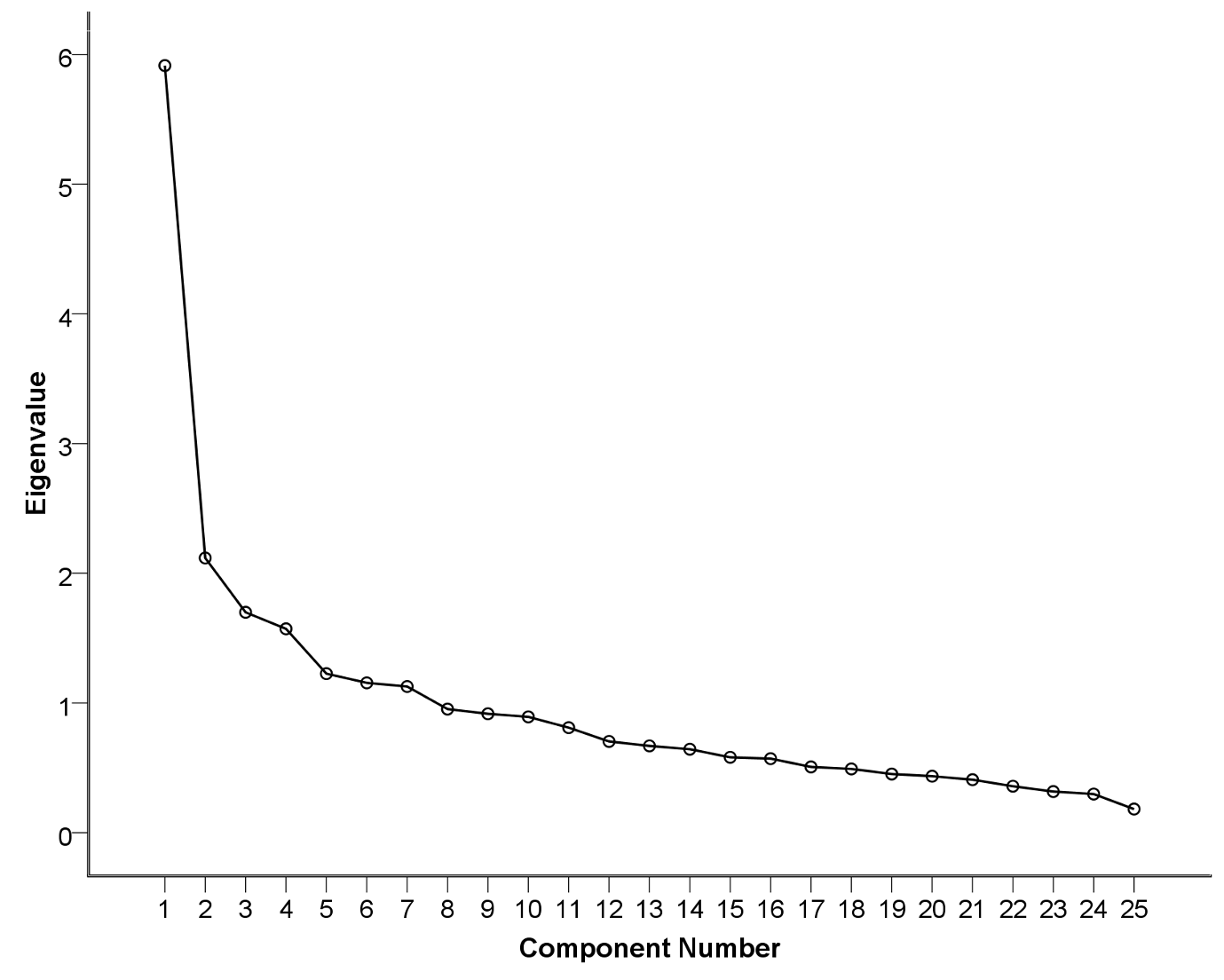


*Figure S1.* Scree plot of the Principal Component Analysis showing the Eigen values of the proposed components.

Table S6.

*Full details of the Principal Component Analysis with varimax rotation of the cognitive tasks. Task order is for display purposes only*

| **Cognitive Task** | **Spatial** | **Verbal** | **IQ/Executive Function** | **Scenes** | **Recognition Memory** | **Semantic Memory** | **Perception** |
| --- | --- | --- | --- | --- | --- | --- | --- |
|  |  |  |  |  |  |  |  |
| Rey-Osterrieth Complex Figure delayed recall | .72 | .26 | .022 | .075 | .023 | -.014 | .18 |
| Paper Folding Test | .72 | .18 | .20 | -.014 | -.047 | .13 | -.073 |
| Navigation | .66 | .11 | .17 | .20 | .17 | -.054 | .10 |
| Object-Place Association Test | .65 | .16 | -.096 | .11 | .11 | -.22 | -.072 |
| Brixton Spatial Anticipation Test | .42 | -.13 | .34 | -.16 | .20 | .18 | -.25 |
| Warrington Recognition Memory Test for Scenes | .54 | .025 | .12 | .13 | .41 | .15 | -.045 |
| Matrix Reasoning | .51 | .069 | .49 | .047 | -.011 | .11 | -.072 |
| Symbol Span | .46 | .29 | .46 | .045 | .065 | .031 | -.005 |
| Rey Auditory Verbal Learning Test delayed recall | .21 | .74 | .063 | .064 | -.002 | .066 | .098 |
| Concrete Verbal Paired Associates delayed recall | .20 | .67 | .20 | .039 | .27 | .25 | -.008 |
| Logical Memory delayed recall | .18 | .66 | .048 | .15 | .005 | -.16 | .032 |
| Verbal Paired Associates delayed recall | .13 | .62 | .17 | .085 | .33 | .061 | -.19 |
| Abstract Verbal Paired Associates delayed recall | .033 | .61 | .46 | -.031 | .13 | .035 | -.043 |
| Digit Span | .16 | .17 | .74 | .046 | -.21 | -.17 | -.098 |
| Full Scale Intelligence Quotient | .024 | .20 | .68 | .075 | .26 | .20 | .20 |
| F-A-S Verbal Fluency | .13 | .11 | .62 | .27 | .062 | .027 | .053 |
| Scene Construction Experiential Index | .16 | .028 | .12 | .87 | .072 | .12 | -.065 |
| Future Thinking Experiential Index | .15 | .006 | .16 | .85 | .091 | .084 | -.023 |
| Autobiographical Memory Internal Details | .024 | .24 | -.001 | .62 | -.010 | .13 | .16 |
| Scene Description | -.094 | .036 | .20 | .37 | .19 | -.24 | .50 |
| Warrington Recognition Memory Test for Words | .16 | .18 | -.084 | .095 | .67 | -.18 | .008 |
| Warrington Recognition Memory Test for Faces | .080 | .15 | .11 | .028 | .79 | .089 | .061 |
| Autobiographical Memory External Details | .060 | -.089 | -.051 | .27 | -.060 | .69 | .17 |
| Dead or Alive Task | -.028 | .15 | .087 | .051 | .028 | .62 | -.067 |
| Boundary Extension | .059 | -.036 | -.30 | -.038 | -.015 | .14 | .84 |
|  |  |  |  |  |  |  |  |
| **Variance explained (Total = 59.24%)** | **12.44** | **10.64** | **9.85** | **9.23** | **6.81** | **5.28** | **5.01** |

Table S7.

*Full details of the Principal Component Analysis with FSIQ removed. Task order is for display purposes only*

| **Cognitive Task** | **Spatial** | **Verbal** | **Scenes** | **IQ/Executive Function** | **Recognition Memory** | **Semantic Memory** | **Perception** |
| --- | --- | --- | --- | --- | --- | --- | --- |
|  |  |  |  |  |  |  |  |
| Rey-Osterrieth Complex Figure delayed recall | .71 | .25 | .062 | .080 | .027 | 0.002 | .19 |
| Paper Folding Test | .70 | .17 | -.036 | .27 | -.038 | .16 | -.052 |
| Navigation | .68 | .13 | .21 | .15 | .13 | -.060 | .071 |
| Object-Place Association Test | .67 | .15 | .10 | -.062 | .086 | -.21 | -.093 |
| Brixton Spatial Anticipation Test | .40 | -.12 | -.16 | .35 | .19 | .21 | -.25 |
| Warrington Recognition Memory Test for Scenes | .56 | .034 | .15 | .097 | .38 | .14 | -.065 |
| Matrix Reasoning | .43 | .065 | .021 | .60 | .050 | .15 | -.005 |
| Symbol Span | .43 | .30 | .056 | .50 | .075 | .037 | .007 |
| Rey Auditory Verbal Learning Test delayed recall | .21 | .73 | .067 | .060 | .011 | .056 | .12 |
| Concrete Verbal Paired Associates delayed recall | .18 | .67 | .034 | .19 | .29 | .26 | .010 |
| Logical Memory delayed recall | .23 | .68 | .16 | -.027 | -.036 | -.16 | -.037 |
| Verbal Paired Associates delayed recall | .11 | .61 | .074 | .19 | .36 | .086 | -.17 |
| Abstract Verbal Paired Associates delayed recall | .009 | .63 | -.013 | .43 | .16 | .027 | -.022 |
| Digit Span | .11 | .20 | .064 | .76 | -.17 | -.16 | -.073 |
| F-A-S Verbal Fluency | .06 | .12 | .28 | .67 | .12 | .040 | 0.12 |
| Scene Construction Experiential Index | .17 | .030 | .86 | .10 | .059 | .13 | -.086 |
| Future Thinking Experiential Index | .15 | .009 | .85 | .15 | .087 | .099 | -.038 |
| Autobiographical Memory Internal Details | .025 | .24 | .62 | -.009 | -.007 | .11 | .18 |
| Scene Description | -.053 | .023 | .42 | .15 | .23 | -.18 | .46 |
| Warrington Recognition Memory Test for Words | .17 | .15 | .080 | -.076 | .69 | -.16 | .010 |
| Warrington Recognition Memory Test for Faces | .085 | .15 | .048 | .073 | .79 | .077 | .073 |
| Autobiographical Memory External Details | .065 | -.090 | .27 | -.075 | -.071 | .69 | .18 |
| Dead or Alive Task | -.038 | .16 | .050 | .061 | .022 | .64 | -.073 |
| Boundary Extension | .045 | -.034 | -.023 | -.035 | -.004 | .11 | .86 |
|  |  |  |  |  |  |  |  |
| **Variance explained (Total = 59.75%)** | **12.47** | **11.04** | **9.73** | **9.09** | **6.98** | **5.37** | **5.07** |

Table S8

*Full details of the regression analyses shown in Figure 1 examining the mediation analyses of the Scene component variables when future thinking is the dependent variable*

|  | **Beta**  **(95% CI)** | **Standardised Beta (95% CI)** | ***t*** | ***p*** |  |
| --- | --- | --- | --- | --- | --- |
| **Future Thinking predicted by Autobiographical Memory** [*F*(1,215 = 38.08, *p* < .001, R^2^ = .15] | | | | | |
|  | | | | | |
| Autobiographical Memory | .39 (.26, .51) | .39 (.26, .51) | 6.17 | < .001 | |
|  |  |  |  |  | |
| **Scene Construction predicted by Autobiographical Memory** [*F*(1,215 = 48.03, *p* < .001, R^2^ = .18] | | | | | |
|  |  |  |  |  | |
| Autobiographical Memory | .36 (.26, .46) | .43 (.33, .53) | 6.93 | < .001 | |
|  |  |  |  |  | |
| **Future Thinking predicted by Scene Construction** [*F*(1,215 = 350.7, *p* < .001, R^2^ = .62] | | | | | |
|  | | | | | |
| Scene Construction | .94 (.84, 1.03) | .79 (.69, .89) | 18.73 | < .001 | |
|  |  |  |  |  | |
| **Autobiographical Memory predicted by Scene Construction** [*F*(1,215 = 48.03, *p* < .001, R^2^ = .18] | | | | | |
|  |  |  |  |  | |
| Scene Construction | .51 (.36, .65) | .43 (.28, .57) | 6.93 | < .001 | |
|  |  |  |  |  | |
| **Future Thinking predicted by Autobiographical Memory and Scene Construction** [*F*(2,214 = 176.9, *p* < .001, Adj. R^2^ = .62] | | | | | |
|  |  |  |  |  | |
| Autobiographical Memory | .063 (-.029, .15) | .063 (-.028, .15) | 1.36 | .18 | |
| Scene Construction | .90 (.80, 1.01) | .76 (.65, .87) | 16.38 | < .001 | |
|  | | | | | |

Table S9

*Full details of the regression analyses shown in Figure 3 examining the mediation analyses of the Scene component variables when future thinking is the independent variable*

|  | **Beta**  **(95% CI)** | **Standardised Beta (95% CI)** | ***t*** | ***p*** |  |
| --- | --- | --- | --- | --- | --- |
| **Autobiographical Memory predicted by Future Thinking** [*F*(1,215 = 38.08, *p* < .001, R^2^ = .15] | | | | | |
|  | | | | | |
| Future Thinking | .39 (.26, .51) | .39 (.26,.51) | 6.17 | < .001 | |
|  |  |  |  |  | |
| **Scene Construction predicted by Future Thinking** [*F*(1,215 = 350.7, *p* < .001, R^2^ = .62] | | | | | |
|  |  |  |  |  | |
| Future Thinking | .66 (.59, .73) | .79 (.72, .86) | 18.73 | < .001 | |
|  |  |  |  |  | |
| **Autobiographical Memory** **predicted by Future Thinking and Scene Construction** [*F*(2,214 = 25.03, *p* < .001, Adj. R^2^ = .18] | | | | | |
|  |  |  |  |  | |
| Future thinking | .14 (-.062, .33) | .14 (-.062, .33) | 1.36 | .18 | |
| Scene Construction | .38 (.15, .62) | .32 (.086, .56) | 3.21 | .0015 | |
|  |  |  |  |  | |
| **Scene Construction** **predicted by Future Thinking and Autobiographical Memory** [*F*(2,214 = 188.1, *p* < .001, Adj. R^2^ = .63] | | | | | |
|  | | | | | |
| Future thinking | .62 (.54, .69) | .73 (.66, .81) | 16.38 | < .001 | |
| Autobiographical Memory | .12 (.047, .19) | .14 (.070, .22) | 3.21 | .0015 | |
|  | | | | | |

Table S10

*Full details of the regression analyses shown in Figure 5 examining the mediation analyses of the scene construction, autobiographical memory and navigation relationships*

|  | **Beta**  **(95% CI)** | **Standardised Beta (95% CI)** | ***t*** | ***p*** |  |
| --- | --- | --- | --- | --- | --- |
| **Navigation predicted by Autobiographical Memory** [*F*(1,215 = 8.92, *p* = .0031, R^2^ = .040] | | | | | |
|  | | | | | |
| Autobiographical memory | .99 (.34, 1.64) | .20 (-.45, .85) | 2.99 | .0032 | |
|  |  |  |  |  | |
| **Scene Construction predicted by Autobiographical Memory** [*F*(1,215 = 48.03, *p* < .001, R^2^ = .18] | | | | | |
|  |  |  |  |  | |
| Autobiographical Memory | .36 (.26, .46) | .43 (.33,.53) | 6.93 | <.001 | |
|  |  |  |  |  | |
| **Navigation predicted by Scene construction** [*F*(1,215 = 19.83, *p* < .001, R^2^ = .084] | | | | | |
|  |  |  |  |  | |
| Scene Construction | 1.71 (.95, 2.47) | .29 (-.47, 1.05) | 4.45 | < .001 | |
|  |  |  |  |  | |
| **Autobiographical Memory predicted by Scene Construction** [*F*(1,215 = 48.03, *p* < .001, R^2^ = .18] | | | | | |
|  |  |  |  |  | |
| Scene Construction | .51 (.36, .65) | .43 (.28, .57) | 6.93 | < .001 | |
|  |  |  |  |  | |
| **Navigation** **predicted by Autobiographical Memory and Scene Construction** [*F*(2,214 = 10.76, *p* < .001, Adj. R^2^ = .083] | | | | | |
|  |  |  |  |  | |
| Autobiographical Memory | .46 (-.25, 1.16) | .092 (-.61, .79) | 1.28 | .20 | |
| Scene Construction | 1.48 (.64, 2.32) | .25 (-.59, 1.09) | 3.49 | < .001 | |
|  | | | | | |

Table S11

*Full details of the regression analyses shown in Figure 7 examining the mediation analyses of the future thinking to navigation relationship with scene construction or autobiographical memory as the mediating variable*

|  | **Beta**  **(95% CI)** | **Standardised Beta (95% CI)** | ***t*** | ***p*** |  |  |
| --- | --- | --- | --- | --- | --- | --- |
| **Navigation predicted by Future thinking** [*F*(1,215 = 14.48, *p* < .001, R^2^ = .063] | | | | | | |
|  | | | | | | |
| Future Thinking | 1.24 (.60, 1.89) | .25 (-.39, .90) | 3.81 | < .001 | | |
|  |  |  |  |  | | |
| **Scene Construction predicted by Future Thinking** [*F*(1,215 = 350.7, *p* < .001, R^2^ = .62] | | | | | | |
|  |  |  |  |  | | |
| Future Thinking | .66 (.59, .73) | .79 (.72, .86) | 18.73 | < .001 | | |
|  |  |  |  |  | | |
| **Autobiographical Memory predicted by Future Thinking** [*F*(1,215 = 38.08, *p* < .001, R^2^ = .15] | | | | | | |
|  |  |  |  |  | | |
| Future Thinking | .39 (.26, .51) | .39 (.26, .51) | 6.17 | < .001 | | |
|  |  |  |  |  | | |
| **Navigation predicted by Future Thinking and Scene Construction** [*F*(2,214 = 10.04, *p* < .001, Adj. R^2^ = .077] | | | | | | |
|  |  |  |  |  | | |
| Future thinking | .29 (-.74, 1.33) | .059 (-.98, 1.09) | .56 | .58 | | |
| Scene Construction | 1.44 (.21, 2.67) | .24 (-.99, 1.48) | 2.30 | .022 | | |
|  |  |  |  |  | | |
| **Navigation** **predicted by Future thinking and Autobiographical Memory** [*F*(2,214 = 8.72, *p* < .001, Adj. R^2^ = .067] | | | | | |  |
|  | | | | | | |
| Future Thinking | 1.01 (.32, 1.71) | .20 (-.49, .90) | 2.87 | .0045 | | |
| Autobiographical Memory | .59 (-.10, 1.29) | .12 (-.57, .82) | 1.69 | .093 | | |
|  | | | | | | |

Table S12

*Details of the pathways within the structural equation model of the Spatial component to navigation relationship, with the Scene component as the mediating variable*

|  | | **Beta**  **(95% CI)** | | **Standardised Beta (95% CI)** | | | ***z*** | ***p*** |
| --- | --- | --- | --- | --- | --- | --- | --- | --- |
| **Spatial Component latent variable predictors** | | | | | | | | |
|  |  | |  | |  |  | | |
| ROCF delayed recall | | 3.87 (3.09, 4.66) | | .68 (.58, .78) | | | 9.67 | < .001 |
| Paper Folding | | 2.59 (2.06, 3.11) | | .68 (.57, .78) | | | 9.61 | < .001 |
| Object-Place Association | | 1.18 (.87, 1.48) | | .55 (.44, .67) | | | 7.62 | < .001 |
| Brixton Spatial Anticipation Test | | .66 (.38, .93) | | .36 (.22, .49) | | | 4.71 | < .001 |
|  | |  | |  | | |  |  |
| **Scene Component latent variable predictors** | | | | | | | | |
|  | |  | |  | | |  |  |
| Scene Construction | | 5.45 (4.67, 6.21) | | .93 (.85, 1.01) | | | 14.05 | < .001 |
| Future Thinking | | 5.87 (4.98, 6.76) | | .84 (.77, .92) | | | 12.90 | < .001 |
| Autobiographical Memory | | 3.21 (2.28, 4.14) | | .46 (.35, .57) | | | 6.77 | < .001 |
|  | |  | |  | | |  |  |
| **Scene Component predicted by the Spatial Component** | | | | | | | | |
|  | |  | |  | | |  |  |
| Spatial Component | | .28 (.1, .46) | | .27 (.11, .43) | | | 3.06 | .002 |
|  | |  | |  | | |  |  |
| **Predictors of Navigation** | | | | | | | | |
|  | | | | | | | | |
| Spatial Component | | 22.71 (17.64, 27.79) | | .64 (.52, .75) | | | 8.77 | < .001 |
| Scene Component | | 4.87 (.47, 9.27) | | .14 (.016, .27) | | | 2.17 | .030 |
|  | |  | |  | | |  |  |

Table S13

*Details of the pathways within the structural equation model of the Spatial component to navigation relationship, with scene construction as the mediating variable*

|  | | **Beta**  **(95% CI)** | | | **Standardised Beta (95% CI)** | | ***z*** | ***p*** |
| --- | --- | --- | --- | --- | --- | --- | --- | --- |
| **Spatial Component latent variable predictors** | | | | | | | | |
|  |  | |  |  | |  | | |
| ROCF delayed recall | | 3.87 (3.09, 4.66) | | | .68 (.58, .78) | | 9.67 | < .001 |
| Paper Folding | | 2.59 (2.06, 3.11) | | | .68 (.57, .78) | | 9.60 | < .001 |
| Object-Place Association | | 1.18 (.87, 1.48) | | | .55 (.43, .67) | | 7.61 | < .001 |
| Brixton Spatial Anticipation Test | | .66 (.39, .93) | | | .36 (.22, .49) | | 4.72 | < .001 |
|  | |  | | |  | |  |  |
| **Scene Construction predicted by the Spatial Component** | | | | | | | | |
|  | |  | | |  | |  |  |
| Spatial Component | | 1.48 (.53, 2.43) | | | .24 (.092, .40) | | 3.05 | .002 |
|  | |  | | |  | |  |  |
| **Predictors of Navigation** | | | | | | | | |
|  | |  | | |  | |  |  |
| Spatial Component | | 22.89 (17.87, 27.92) | | | .64 (.53, .75) | | 8.92 | < .001 |
| Scene Construction | | .79 (.096, 1.49) | | | .13 (.016, .25) | | 2.23 | .026 |
|  | |  | | |  | |  |  |

Table S14

*Details of the pathways within the structural equation model of the Spatial component to navigation relationship, with autobiographical memory as the mediating variable*

|  | | **Beta**  **(95% CI)** | | **Standardised Beta (95% CI)** | | ***z*** | ***p*** |
| --- | --- | --- | --- | --- | --- | --- | --- |
| **Spatial Component latent variable predictors** | | | | | | | |
|  |  | |  | |  |  | |
| ROCF delayed recall | | 3.86 (3.08, 4.65) | | .68 (.58, .78) | | 9.63 | < .001 |
| Paper Folding | | 2.59 (2.06, 3.12) | | .68 (.57, .78) | | 9.61 | < .001 |
| Object-Place Association | | 1.17 (.87, 1.48) | | .55 (.43, .67) | | 7.60 | < .001 |
| Brixton Spatial Anticipation Test | | .67 (.39, .94) | | .36 (.22, .50) | | 4.77 | < .001 |
|  | |  | |  | |  |  |
| **Autobiographical Memory predicted by the Spatial Component** | | | | | | | |
|  | |  | |  | |  |  |
| Spatial Component | | .81 (-.34, 1.96) | | .11 (-.046, .27) | | 1.38 | .17 |
|  | |  | |  | |  |  |
| **Predictors of Navigation** | | | | | | | |
|  | |  | |  | |  |  |
| Spatial Component | | 23.57 (18.67, 28.48) | | .66 (.55, .76) | | 9.42 | < .001 |
| Autobiographical Memory | | .62 (.057, 1.19) | | .13 (.012, .24) | | 2.16 | .031 |
|  | |  | |  | |  |  |

Table S15

*Details of the pathways within the structural equation model of the Spatial component to navigation relationship, with future thinking as the mediating variable*

|  | | **Beta**  **(95% CI)** | **Standardised Beta (95% CI)** | | ***z*** | ***p*** |
| --- | --- | --- | --- | --- | --- | --- |
| **Spatial Component latent variable predictors** | | | | | | |
|  |  |  |  |  | | |
| ROCF delayed recall | | 3.87 (3.08, 4.65) | .68 (.58, .78) | | 9.65 | < .001 |
| Paper Folding | | 2.59 (2.06, 3.11) | .68 (.57, .78) | | 9.61 | < .001 |
| Object-Place Association | | 1.18 (.88, 1.48) | .55 (.44, .67) | | 7.65 | < .001 |
| Brixton Spatial Anticipation Test | | .66 (.38, .93) | .36 (.22, .49) | | 4.71 | < .001 |
|  | |  |  | |  |  |
| **Future Thinking predicted by the Spatial Component** | | | | | | |
|  | |  |  | |  |  |
| Spatial Component | | 1.74 (.61, 2.87) | .24 (.089, .39) | | 3.01 | .003 |
|  | |  |  | |  |  |
| **Predictors of Navigation** | | | | | | |
|  | |  |  | |  |  |
| Spatial Component | | 23.26 (18.18, 28.33) | .65 (.54, .76) | | 8.98 | < .001 |
| Future Thinking | | .47 (-.12, 1.06) | .094 (-.025, .21) | | 1.55 | .12 |
|  | |  |  | |  |  |

**Control mediation analyses between the Verbal Memory component and the tasks of the Scene component**

The Scene component of the PCA contained tasks that were scored from open ended verbal descriptions. As such, verbal task demands - be that narrative style, verbal ability and so forth - or similarities in scoring across the tasks could be candidate processes linking scene construction, autobiographical memory and future thinking. As we detail in the main text, we do not believe this to be the case due to the pattern of results that emerged. However, to further examine the potential involvement of verbal processing, we also ran a series of control mediation analyses looking at the effects of the Verbal Memory component (as a proxy for verbal ability) on the tasks of the Scene component.

We did this by employing the same methodology as when relating the Spatial and Scene components to navigation. In short, using SEM, a latent variable was used to represent the Verbal Memory component. The Verbal Memory latent variable was comprised of the tasks identified by the PCA, namely: Concrete Verbal Paired Associates, WMS Verbal Paired Associates, RAVLT, Abstract Verbal Paired Associates and the Logical Memory test.

Figure S2 shows the SEMs of the relationships between the Verbal Memory component and each of autobiographical memory, future thinking and scene construction when mediated by the other tasks of the Scene component (i.e. scene construction, autobiographical memory or future thinking). The latent variable (Verbal Memory) is shown in a circle, the observed variables (the cognitive tasks) in rectangles. The numerical values represent the standardised coefficients of the path in question. For all models, overall model fit was good, in line with published recommendations [a: *χ^2^*(13) = 18.52, *p* = .14; CFI = .98; TLI = .97; RMSEA = .044 (90% CI: 0, .086); SRMR = .037; b: *χ^2^*(13) = 14.23, *p* = .36; CFI = .997; TLI = .996; RMSEA = .021 (90% CI: 0, .072); SRMR = .029; c: *χ^2^*(13) = 18.52, *p* = .14; CFI = .98; TLI = .97; RMSEA = .044 (90% CI: 0, .086); SRMR = .037; d: *χ^2^*(13) = 18.14, *p* = .15; CFI = .98; TLI = .97; RMSEA = .043 (90% CI: 0, .085); SRMR = .036; e: *χ^2^*(13) = 14.23, *p* = .36; CFI = .997; TLI = .996; RMSEA = .021 (90% CI: 0, .072); SRMR = .029; f: *χ^2^*(13) = 18.14, *p* = .15; CFI = .98; TLI = .97; RMSEA = .043 (90% CI: 0, .085); SRMR = .036].

Of key relevance to our question, the influence of the Verbal Memory component was either fully or partially mediated in all the models. This suggests that the results reported in the main text showing the relationships between scene construction, autobiographical memory and future thinking cannot simply be explained by verbal ability.


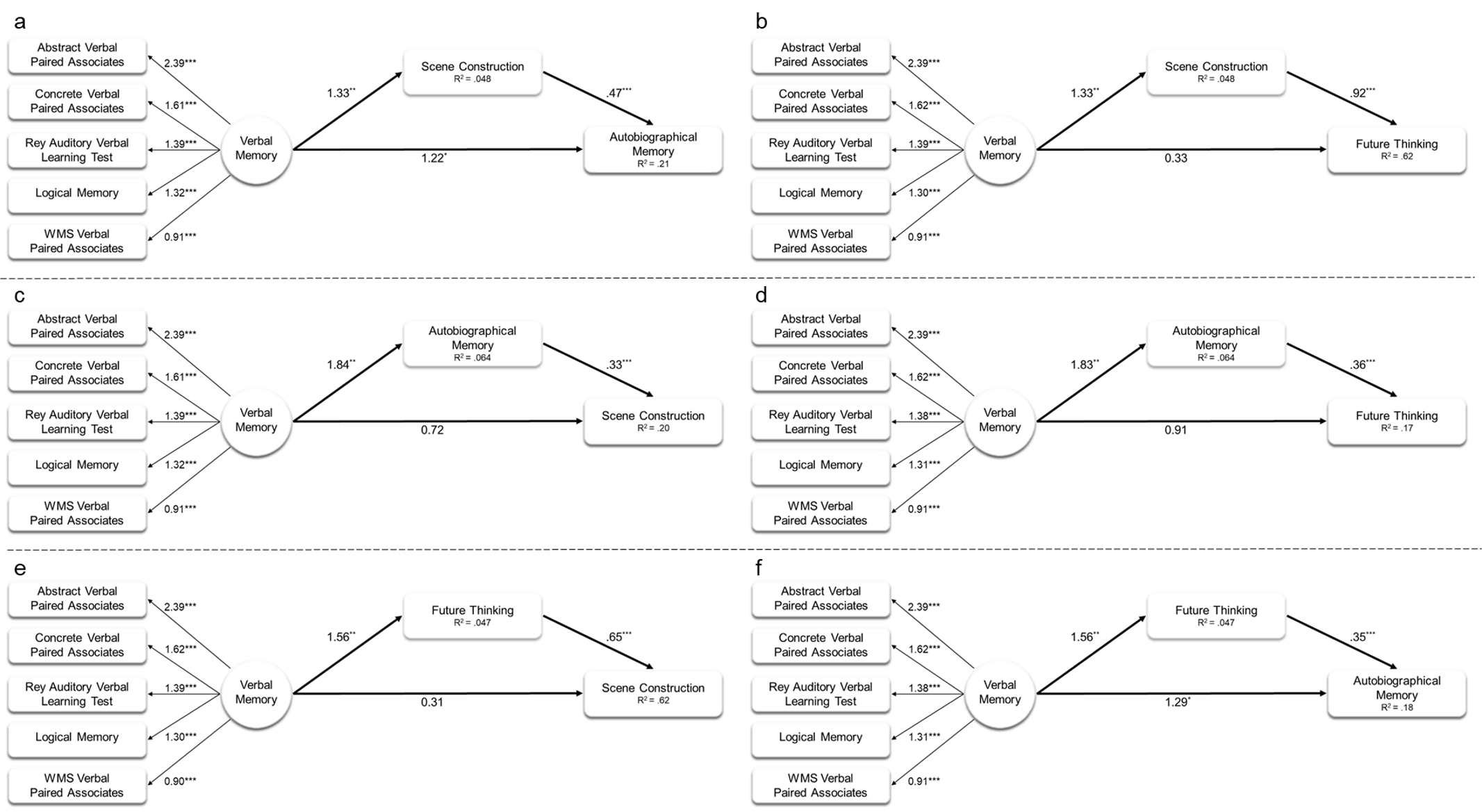


*Figure S2*. Structural equation models of the mediation effects of scene construction, autobiographical memory or future thinking on the Verbal Memory component to scene construction, autobiographical memory or future thinking relationship. The darker arrows show the main paths of interest, the lighter arrows show the links between the individual observed variables and the latent variable (Verbal Memory). The R2 values represent the proportion of variance explained by the main paths of interest (i.e. the dark arrows). Numerical values linked with a pathway represent standardized path coefficients. *p < .05, **p < .01, ***p < .001.

**Control mediation analyses using the delayed recall of the ROCF as the mediator variable between the tasks of the Scene component**

A potential criticism of our previous analyses is that we tested the influence of memory on the relationship between scene construction, autobiographical memory and future thinking using a memory task that relied on verbal output. To address this issue, we also examined the relationships between scene construction, autobiographical memory and future thinking with a nonverbal memory task (the delayed recall of the ROCF) as the mediator.

First, we ascertained whether mediation by the ROCF was possible. As detailed in the main text, according to the steps outlined by Baron & Kenny (1986), for mediation to occur, the independent variable must predict the mediator variable. However, while scene construction and future thinking predicted the ROCF, autobiographical memory did not (see Table S16). As such, while it has also been suggested that an initial direct relationship between the independent variable (autobiographical memory) and the dependent variable (ROCF) is not required in the presence of a strong a priori belief of a small effect size (e.g. Shrout & Bolger, 2002), given the substantial sample size of the current study and the observed effect size of 0.0047 we deemed that the ROCF could not, mediate the relationships between autobiographical memory and scene construction, and autobiographical memory and future thinking. Moreover, as no relationship was found between the ROCF and autobiographical memory, the ROCF could also not mediate between scene construction and autobiographical memory, or future thinking and autobiographical memory. As such, there were only two possible relationships that the ROCF could theoretically mediate – the scene construction to future thinking relationship, and the future thinking to scene construction relationship. However, mediation analyses found no effect of the ROCF on either (Tables S16-17). The ROCF did not, therefore, mediate between any of the tasks of the Scene component.

Table S16

*Full details of the regression analyses examining the mediation analyses using the delayed recall of the ROCF as the mediator variable between the tasks of the Scene component*

|  | **Beta**  **(95% CI)** | **Standardised Beta (95% CI)** | ***t*** | ***p*** |  |  |
| --- | --- | --- | --- | --- | --- | --- |
| **ROCF delayed recall predicted by Scene Construction** [*F*(1,215 = 8.67, *p* = .0036, R^2^ = .039] | | | | | | |
|  | | | | | | |
| Scene Construction | .18 (.061, .31) | .20 (.073, .32) | 2.95 | .0036 | | |
|  |  |  |  |  | | |
| **ROCF delayed recall predicted by Autobiographical Memory** [*F*(1,215 = 1.02, *p* = .31, R^2^ = .0047] | | | | | | |
|  |  |  |  |  | | |
| Autobiographical Memory | .054 (-.05, .16) | .69 (-.38, .17) | 1.09 | .32 | | |
|  |  |  |  |  | | |
| **ROCF delayed recall predicted by Future Thinking** [*F*(1,215 = 6.10, *p* = .014, R^2^ = .023] | | | | | | |
|  |  |  |  |  | | |
| Future Thinking | .13 (.026, .24) | .17 (.061, .27) | 2.47 | .014 | | |
|  |  |  |  |  | | |
| **Scene Construction predicted by Future Thinking and ROCF delayed recall** [*F*(2,214 = 177.9, *p* < .001, Adj. R^2^ = .62] | | | | | | |
|  |  |  |  |  | | |
| Future thinking | .65 (-.74, 1.33) | .78 (.71, .85) | 18.27 | < .001 | | |
| Rey Figure | .072 (-.017, .16) | .067 (-.021, .16) | 1.60 | .11 | | |
|  |  |  |  |  | | |
| **Future Thinking** **predicted by Scene Construction and ROCF delayed recall** [*F*(2,214 = 174.6, *p* < .001, Adj. R^2^ = .62] | | | | | |  |
|  | | | | | | |
| Scene Construction | .93 (.83, 1.03) | .79 (.68, .89) | 18.27 | < .001 | | |
| Rey Figure | .015 (-.093, .12) | .011 (-.096, .12) | .27 | .79 | | |
|  | | | | | | |

Table S17

*Mediation analyses of the Scene component variables with the delayed recall of the ROCF as the mediator variable*

|  | **Beta (95% CI)** | ***p*** | **Sensitivity (ρ)** |
| --- | --- | --- | --- |
| a | | | |
| **Future Thinking to Scene Construction, mediated by ROCF delayed recall** | | | |
| Indirect Effect (ACME) | .0094 (-.0019, .03) | .12 | .1 |
| Direct Effect (ADE) | .65 (.59, .72) | < .001 | -.95 |
| Total | .66 (.60, .73) | < .001 | n/a |
|  |  |  |  |
| b |  |  |  |
| **Scene Construction to Future Thinking, mediated by ROCF delayed recall** | | | |
| Indirect Effect (ACME) | .0027 (-.018, .03) | .79 | 0.0 |
| Direct Effect (ADE) | .93 (.83, 1.03) | < .001 | -.95 |
| Total | .93 (.84, 1.03) | < .001 | n/a |
|  |  |  |  |
